# Arginine Metabolic Disruption Impairs Hair Regeneration via ROS‐Mediated Inactivation of mTOR Signaling in Androgenetic Alopecia

**DOI:** 10.1002/advs.202504579

**Published:** 2025-08-07

**Authors:** Shixin Duan, Guo Li, Yanji Chu, Junbo Zhang, Li Yang, Yujin Zhang, Fangfen Liu, Jiayun Li, Mengting Chen, Ben Wang, Zhixiang Zhao, Wei Shi, Yiya Zhang, Guangtong Deng, Xinwei Kuang, Hongfu Xie, Yufan Cheng, Zhili Deng, Ji Li, Yan Tang

**Affiliations:** ^1^ Department of Dermatology Xiangya Hospital Central South University Changsha Hunan 410008 China; ^2^ Hunan Key Laboratory of Aging Biology Xiangya Hospital Central South University Changsha Hunan 410008 China; ^3^ National Clinical Research Center for Geriatric Disorders Xiangya Hospital Central South University Changsha Hunan 410008 China; ^4^ FuRong Laboratory Changsha 410008 China; ^5^ Department of Dermatology The Second Hospital of Hunan University of Chinese Medicine Changsha Hunan 410005 China; ^6^ Hunan Key Laboratory of Skin Cancer and Psoriasis Hunan Engineering Research Center of Skin Health and Disease Xiangya Hospital Central South University Changsha 410008 China; ^7^ Department of Dermatology Guangdong Provincial People's Hospital Guangzhou 510080 China

**Keywords:** androgenetic alopecia (AGA), arginine metabolism, hair regeneration, therapeutic strategies

## Abstract

Androgenetic alopecia (AGA), a pervasive hair loss disorder, lacks effective therapies due to incomplete pathogenic understanding. Growing evidence suggests a connection between AGA and metabolic disorders. Leveraging unbiased serum metabolomics, a strikingly differentiated metabolic signature in AGA patients compared to healthy controls is identified, with arginine deficiency exhibiting the most pronounced reduction among all amino acids. Concomitant downregulation of the arginine transporter SLC7A1 and upregulation of arginine catabolic enzyme ARG2 in balding HFs are further identified, collectively driving localized arginine scarcity through impaired uptake and accelerated catabolism. This metabolic perturbation triggers pathological reactive oxygen species (ROS) accumulation in hair follicles (HFs), which, in turn, inhibits mTOR signaling and impairs HF regeneration. Conversely, arginine restoration via exogenous supplementation or inhibiting arginine‐to‐ornithine conversion with ARG2 siRNA rescues hair growth in both murine AGA model and cultured human HFs. Most importantly, a microneedle‐based delivery system for targeted dermal arginine replenishment demonstrates robust therapeutic efficacy in humanized AGA models. This work establishes arginine insufficiency as a core pathogenic driver in AGA and validates localized metabolic correction as a promising clinical strategy.

## Graphical abstract

Arginine deficiency accelerates androgenetic alopecia. Serum metabolome shows reduced circulating arginine, while local metabolic disruption depletes follicular reserves, triggering reactive oxygen species (ROS) accumulation and mTOR suppression that induce hair miniaturization. Topical arginine delivery via microneedles reverses this process, demonstrating therapeutic potential.

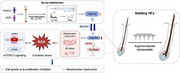

## 1 Introduction

Androgenetic alopecia (AGA) is the most common form of non‐scarring hair loss, affecting up to 74.8% of men and a significant proportion of women worldwide.^[^ ^1^, ^2^, ^3^, ^4^ ^]^ It is characterized by progressive follicular miniaturization, particularly in the frontal and vertex regions, leading to substantial psychological and social consequences.^[^ ^5^, ^6^, ^7^ ^]^ While AGA has long been associated with genetic susceptibility and androgenic signaling, particularly the effects of dihydrotestosterone (DHT), its underlying molecular mechanisms remain poorly understood, and effective treatments are still limited.^[^ ^8^, ^9^, ^10^, ^11^, ^12^ ^]^ Current therapies, such as finasteride and minoxidil, yield only modest results, underscoring the need for novel therapeutic approaches.^[^ ^13^, ^14^, ^15^ ^]^

Recent studies have established a strong link between AGA and various metabolic disorders.^[^ ^16^, ^17^, ^18^ ^]^ Individuals with AGA are more likely to develop conditions such as metabolic syndrome, insulin resistance, and type 2 diabetes.^[^ ^17^ ^]^ Notably, early‐onset AGA (before age 35) has been identified as a potential clinical marker of insulin resistance.^[^ ^19^ ^]^ Moreover, obesity has been shown to induces AGA‐like hair follicle miniaturization in mouse models, further suggesting that metabolic dysregulation plays a critical role in AGA pathogenesis.^[^ ^20^ ^]^ Despite these clinical correlations, the specific mechanisms linking metabolic disturbances to AGA remain largely unexplored.

Metabolic reprogramming, a hallmark of various diseases, governs critical cellular processes such as growth, differentiation, and survival.^[^ ^21^, ^22^, ^23^, ^24^, ^25^ ^]^ Among metabolic alterations observed across pathological conditions, amino acid imbalances are increasingly recognized as critical drivers of cellular dysfunction.^[^ ^26^, ^27^, ^28^ ^]^ Specifically, arginine has emerged as a critical metabolic node due to its central role in protein synthesis, nitric oxide (NO) production, and redox regulation.^[^ ^29^, ^30^, ^31^, ^32^, ^33^ ^]^ For instance, arginine metabolism has been shown to influence immune responses and tumor progression.^[^ ^34^, ^35^, ^36^, ^37^ ^]^ In diseases such as hepatocellular carcinoma (HCC), elevated arginine levels support rapid cell proliferation, and targeting arginine metabolism has become a potential therapeutic strategy.^[^ ^38^, ^39^, ^40^ ^]^ Conversely, reduced arginine availability impairs NO production, contributing to endothelial dysfunction and hypertension.^[^ ^29^, ^41^, ^42^ ^]^ Additionally, Sirt3, a mitochondrial deacetylase, has been shown to protect endothelial cells from atherosclerosis by regulating ASS1‐mediated arginine biosynthesis.^[^ ^41^ ^]^ These findings suggest that modulating arginine metabolism may offer therapeutic potential for a range of diseases.

This study reveals emergent metabolic alterations in AGA patients, with pronounced arginine deficiency in balding HFs constituting a previously unrecognized pathogenic axis. We establish that arginine scarcity—driven by systemic depletion and localized dysregulation (impaired uptake and accelerated catabolism)—directly drives hair growth suppression through ROS‐mediated mTOR pathway inhibition. Critically, arginine restoration via supplementation or ARG2 silencing reverses HF regression in DHT‐induced AGA murine models and primary human HF cultures. Moreover, microneedle‐enabled targeted arginine delivery to the dermis elicits potent hair regeneration in both murine and humanized AGA models, validating this localized metabolic reprogramming as a translatable therapeutic strategy.

## 2 Results

### 2.1 Metabolic Alterations in AGA Patients Compared to Healthy Controls

**Figure 1:**
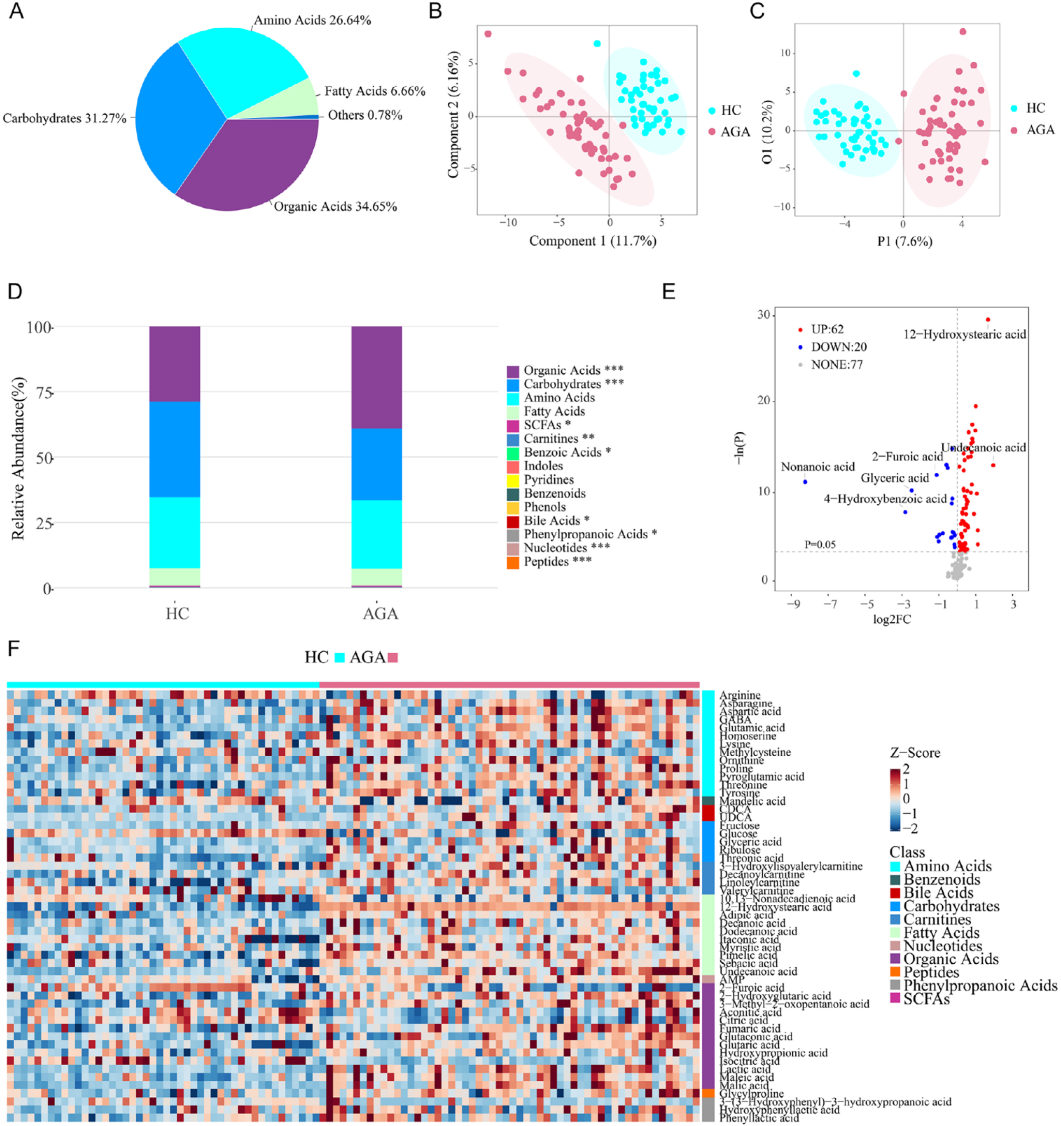
Serum metabolic profiling reveals distinct signatures in androgenetic alopecia (AGA) patients. Targeted metabolomics analysis comparing serum samples from healthy controls (*n* = 52) and AGA patients (*n* = 48): A) Metabolite class distribution. B) Partial least squares‐discriminant analysis (PLS‐DA) score plot. C) Orthogonal PLS‐DA (OPLS‐DA) score plot. D) Relative abundance of metabolite classes. E) Volcano plot of altered metabolites (significance threshold: *P* = 0.05; red: upregulated, blue: downregulated). F) Heatmap of 54 differentially abundant metabolites.

To examine potential metabolites implicated in the development of androgenetic alopecia (AGA), we conducted a comprehensive metabolomics analysis on a cohort consisting of 48 AGA patients and 52 age‐ and sex‐matched healthy controls. A total of 306 metabolites were quantitatively assessed using ultra‐performance liquid chromatography coupled with tandem mass spectrometry (UPLC‐MS/MS), ultimately identifying 159 metabolites, which include carbohydrates, amino acids, fatty acids, organic acids, and other compounds (**Figure** **1**A). Principal component analysis (PCA) and orthogonal partial least squares discriminant analysis (OPLS‐DA) clearly distinguished AGA patients from healthy controls (Figure 1B,C). Further analysis of metabolite abundance showed an increased proportion of organic acid metabolites and a decrease in carbohydrates in AGA patients compared to the healthy controls (Figure 1D). A total of 82 differentially expressed metabolites (DEMs) were identified, primarily originating from amino acids (24%), organic acids (24%), and fatty acids (18.5%) (Figure 1E,F).

### 2.2 Impaired Arginine Metabolism in AGA Patients

**Figure 2:**
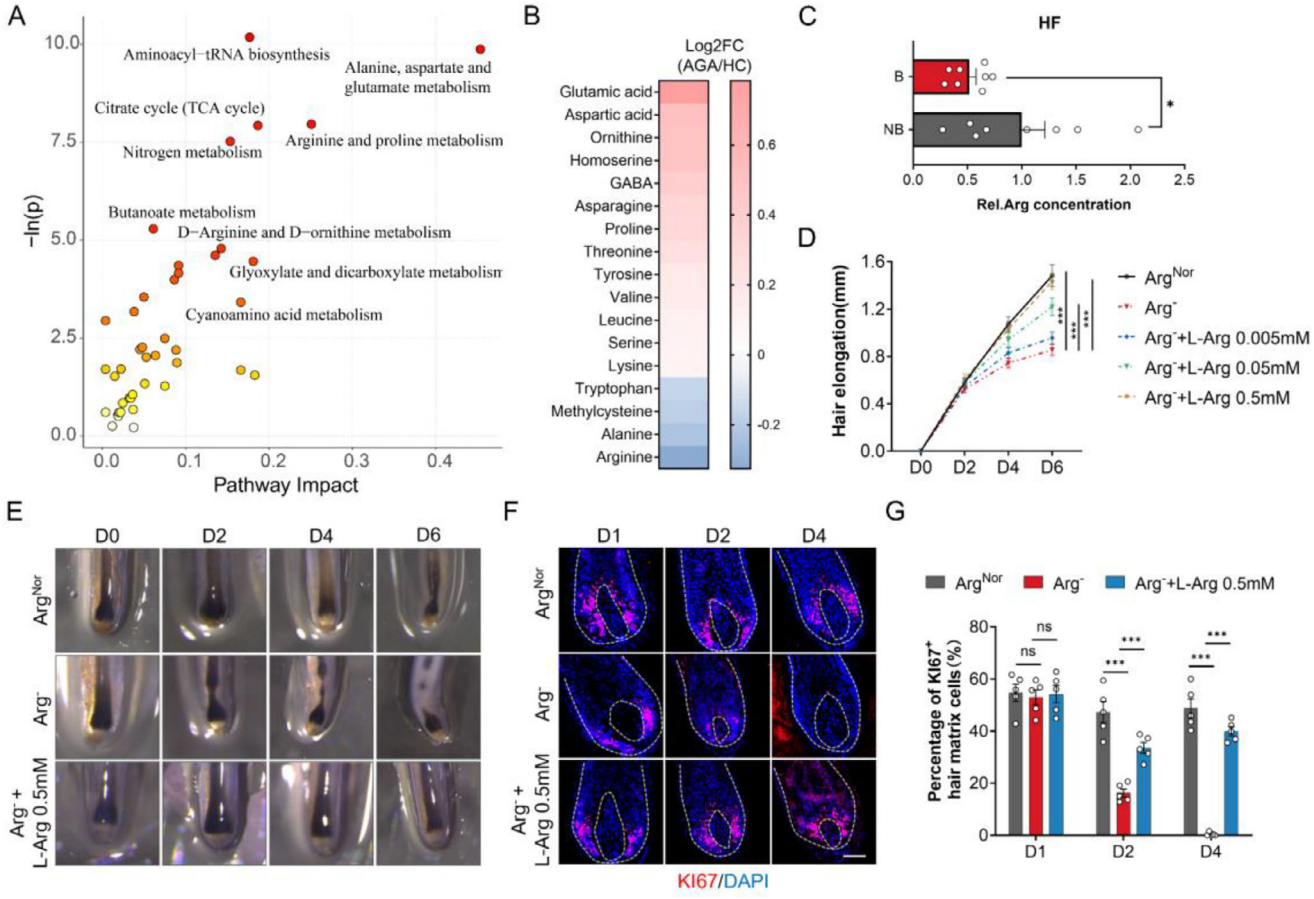
Arginine is essential for human HF growth ex vivo. A) Bubble plot of metabolomic pathway analysis highlighting dysregulated amino acid metabolism in AGA patients. B) Differential abundance of amino acids identified in metabolomic profiling. C) Reduced arginine concentration in balding (B) versus non‐balding (NB) scalp HFs from androgenetic alopecia (AGA) patients (*n* = 8 patients; 30 HFs/patient). D) Hair shaft elongation under normal arginine (Arg^Nor^), arginine‐deficient (Arg^‐^), or Arg^‐^ supplemented with L‐arginine (0.005–0.5 mm) medium (*n* = 24/23/29/31/25 HFs from 3 donors). E) Time‐course images (days 0/2/4/6) of HF lower bulbs cultured in medium of Arg^Nor^, Arg^−^, or Arg^−^ + 0.5 mm L‐arginine. F) KI67 immunostaining of HFs. G) Quantification of the percentage of KI67^+^ matrix cells (*n* = 5 HFs per group). The white curve outlines the general contour of the selected hair bulb and dermal papilla (DP) (F). Data are presented as means ± SEM. Statistical significance was determined by one‐way ANOVA with Tukey's post hoc test (G) and two‐way ANOVA with Dunnett's post hoc test (D). *P < 0.05, **P < 0.01, ***P < 0.001. Scale bar: 50 µm.

Next, we performed pathway and enrichment analyses to further explore the metabolic changes in AGA patients. Our results revealed significant alterations in amino acid metabolism, particularly in the metabolism of aspartate, arginine, and proline (**Figure** **2**A). Statistical analysis of significantly altered amino acids in AGA patient serum revealed glutamic acid and aspartic acid as the most prominently upregulated amino acid, while arginine and alanine exhibited the most substantial downregulation (Figure 2B).

We subsequently employed cultured human HFs to screen the functional impact of these dysregulated amino acids on hair growth. While alanine, aspartic acid, and glutamic acid had minimal effects on HF growth (Figure S1A–C, Supporting Information), arginine significantly promoted hair growth (Figure S1D,E, Supporting Information). We further assessed the proliferation of hair matrix cells, which contribute to hair shaft formation, under arginine treatment using Ki67 staining. Arginine was found to promote hair matrix cell proliferation in a dose‐dependent manner (Figure S1F,G, Supporting Information).

This evidence focused our investigation on arginine. To elucidate the exact local state of arginine metabolism, we compared the mRNA expression levels of genes related to arginine recycling and transport in balding (B) and non‐balding (NB) scalps via RNA‐sequencing. The results showed upregulation of the arginine catabolic enzymes ARG1 and ARG2, coupled with downregulation of key arginine transporters, including the primary transporter SLC7A1, in the balding scalps (Figure S2A, Supporting Information). These findings suggest a potential dysfunction in arginine metabolism, leading to reduced arginine availability in balding hair follicles (HFs).

To validate this hypothesis, we measured local arginine concentrations in non‐balding and balding HFs, observing a significant reduction in arginine levels in balding HFs (Figure 2C). Western blot (WB) and immunofluorescence analyses further confirmed the dysregulation of arginine metabolism, showing upregulation of ARG2 and downregulation of SLC7A1 at the protein level in balding HFs (Figure S2B–G, Supporting Information). To visualize arginine absorption, we synthesized arginine conjugated with FITC for ex vivo imaging. This experiment revealed a significant reduction in arginine uptake in balding HFs (Figure S2H, Supporting Information). Taken together, these results provide evidence of arginine deficiency, potentially due to impaired uptake and enhanced catabolism in the balding HFs of AGA patients.

### 2.3 Arginine is Essential for Hair Growth

To determine the potential role of arginine deficiency on the pathogenesis of AGA, we cultured HFs in medium deprived of arginine. As anticipated, arginine depletion severely impaired HF elongation, an effect that could be reversed by arginine replenishment in a dose‐dependent manner (Figure 2D). Notably, two‐day arginine depletion induced significant morphological alterations in HFs, characterized by shaft narrowing and thinning of the suprabulbar hair matrix. By day 4, further progression manifested as pronounced hair shaft constriction and bulb detachment, indicating arrested hair formation (Figure 2E). Consistent with these structural perturbations, KI67 and pHH3 immunostaining confirmed that arginine deprivation reduced the proliferation of hair matrix cells (Figure 2F,G and Figure S3A,B, Supporting Information). Moreover, primary human HF outer root sheath (ORS) cells were established as an ex vivo cell model for HF keratinocyte functionality analysis. Both EDU incorporation assays and colony‐forming assay further validated that arginine deprivation potently suppressed HF cellular proliferation (Figure S3C—E, Supporting Information). Conversely, TUNEL staining revealed negligible apoptosis in the hair matrix following arginine depletion (Figure S3F,G, Supporting Information), indicating that proliferative impairment—rather than apoptotic induction—drives the observed growth suppression.

**Figure 3:**
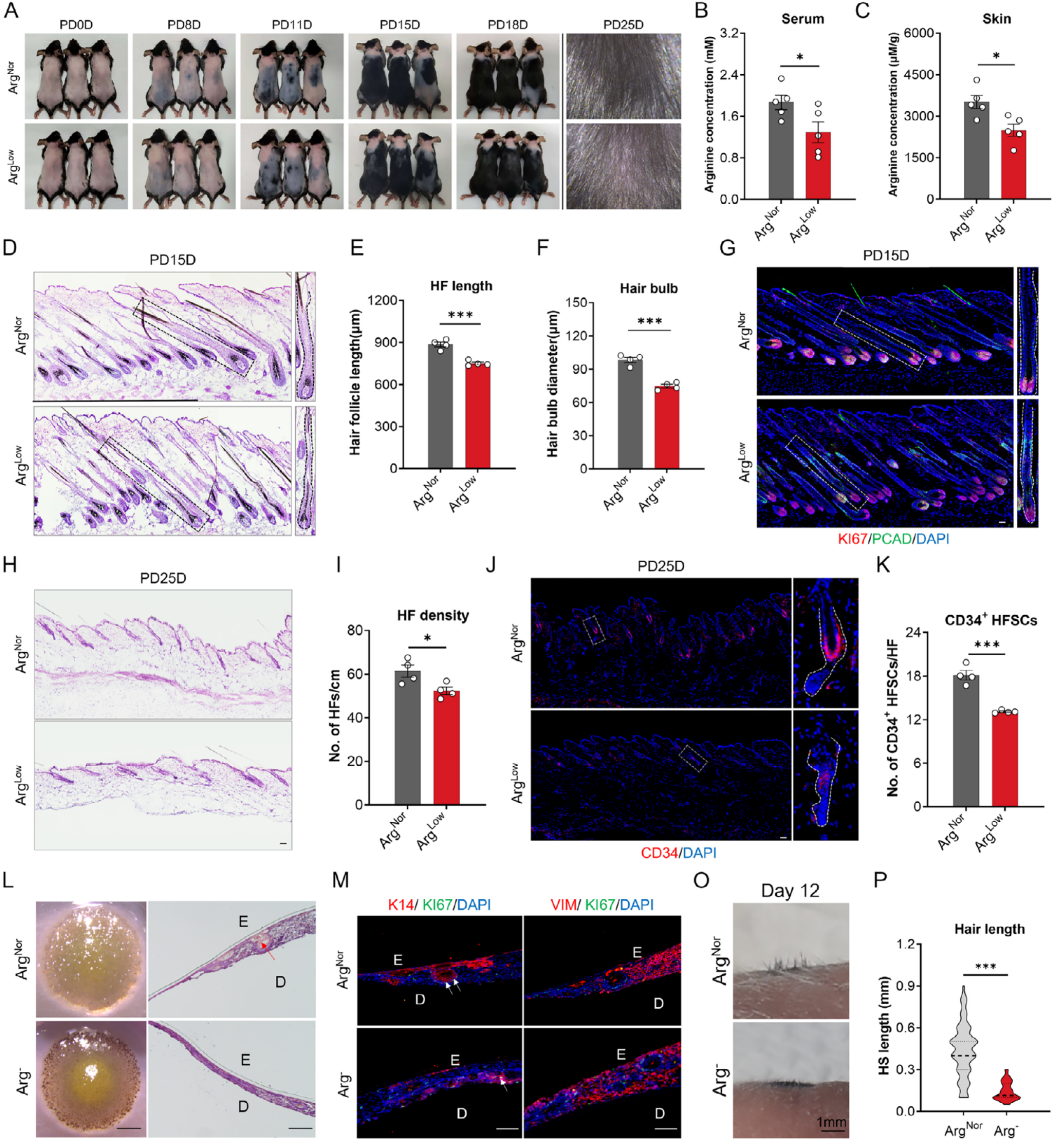
Arginine deficiency impairs hair regeneration in vivo. A) Haircoat recovery in mice fed with normal diet and low‐arginine‐diet, captured at post‐depilation (PD) days 0, 8, 11, 15, and 18. Representative magnified images of mice neck regions at PD25 are shown (*n* = 5 mice per group). Arg^Nor^, normal‐arginine‐diet (6.3 g kg^−1^)‐fed mice; Arg^Low^, low‐arginine‐diet (3 g kg^−1^)‐fed mice. B) Serum and C) skin arginine quantification (*n* = 5 mice per group). D) Hematoxylin and eosin (H&E) staining of dorsal skin sections from mice at PD15D. Quantification of E) HF length and F) hair bulb diameter at PD15D. G) Co‐immunostaining of KI67 and PCAD in skin sections. H) H&E‐stained sections and I) quantification of HF density (I) at PD25D. J) CD34 staining and K) quantification of CD34^+^ hair follicle stem cells (HFSCs) at PD25D (*n* = 4 mice per group). L) Representative morphology and H&E staining of skin organoids under Arg^Nor^ or arginine‐deprived (Arg^−^) culture. M) Co‐immunostaining of KI67 and epithelial marker K14 or mesenchymal marker vimentin in organoid sections. N) Hair regeneration 12 d post‐organoid transplantation onto nude mice. O) Quantified regenerated hair length in two groups. The white/black dashed square indicates the magnified area (G, J). The white/black curve outlines the general contour of the selected hair follicles (D, G). Data are presented as means ± SEM. Statistical significance was determined by student t'test. *P < 0.05, **P < 0.01, ***P < 0.001. Scale bar = 1 mm in O and 50 µm in others.

To evaluate the in vivo impact of arginine bioavailability on hair regeneration, mice were subjected to either normal‐arginine (Arg^Nor^) or 50% arginine‐reduced (Arg^Low^) diets. The Arg^Low^ mice exhibited impaired hair regrowth following depilation‐induced hair regeneration and demonstrated visible hair loss (**Figure** **3**A). The detection of arginine concentration in the circulation and skin of the mice from two groups, confirmed a reduction in arginine levels in the Arg^Low^ group (Figure 3B,C). Histological analysis via HE and Ki67 staining revealed compromised HF regeneration characterized by shortened HF length, diminished hair bulb size, and reduced proliferating matrix cells (Figure 3D–G). Notably, morphometric assessment further identified a slight reduction in HF density and a substantial depletion of CD34^+^ HF stem cells (HFSCs) in Arg^Low^ mice after one hair cycle (Figure 3H–K), suggesting arginine sufficiency as physiologically essential for maintaining the HFSC reservoir necessary for cyclic HF regeneration.

In a skin organoid model, which provides a robust platform for studying HF biology,^[^ ^43^, ^44^ ^]^ our findings demonstrated that arginine deprivation significantly compromised organoid morphogenesis, inducing heterogeneous epithelial stratification characterized by abnormally thickened central regions and attenuated peripheral zones (Figure 3L). Microscopic analysis further revealed that arginine deprivation impeded K14^+^ epithelial aggregate formation and suppressed cellular proliferation, while sparing mesenchymal cell population (Figure 3M). Crucially, when transplanted into nude mice, organoids preconditioned under arginine‐deprived medium yielded substantially diminished hair growth at day 12 post‐grafting, manifesting as reduced hair density and abbreviated shaft length (Figure 3N,O).

Overall, these results collectively underscore the indispensable role of arginine bioavailability on regeneration capacity of HFs.

### 2.4 Deficiency of Arginine Induces ROS Accumulation in Hair Follicles

**Figure 4:**
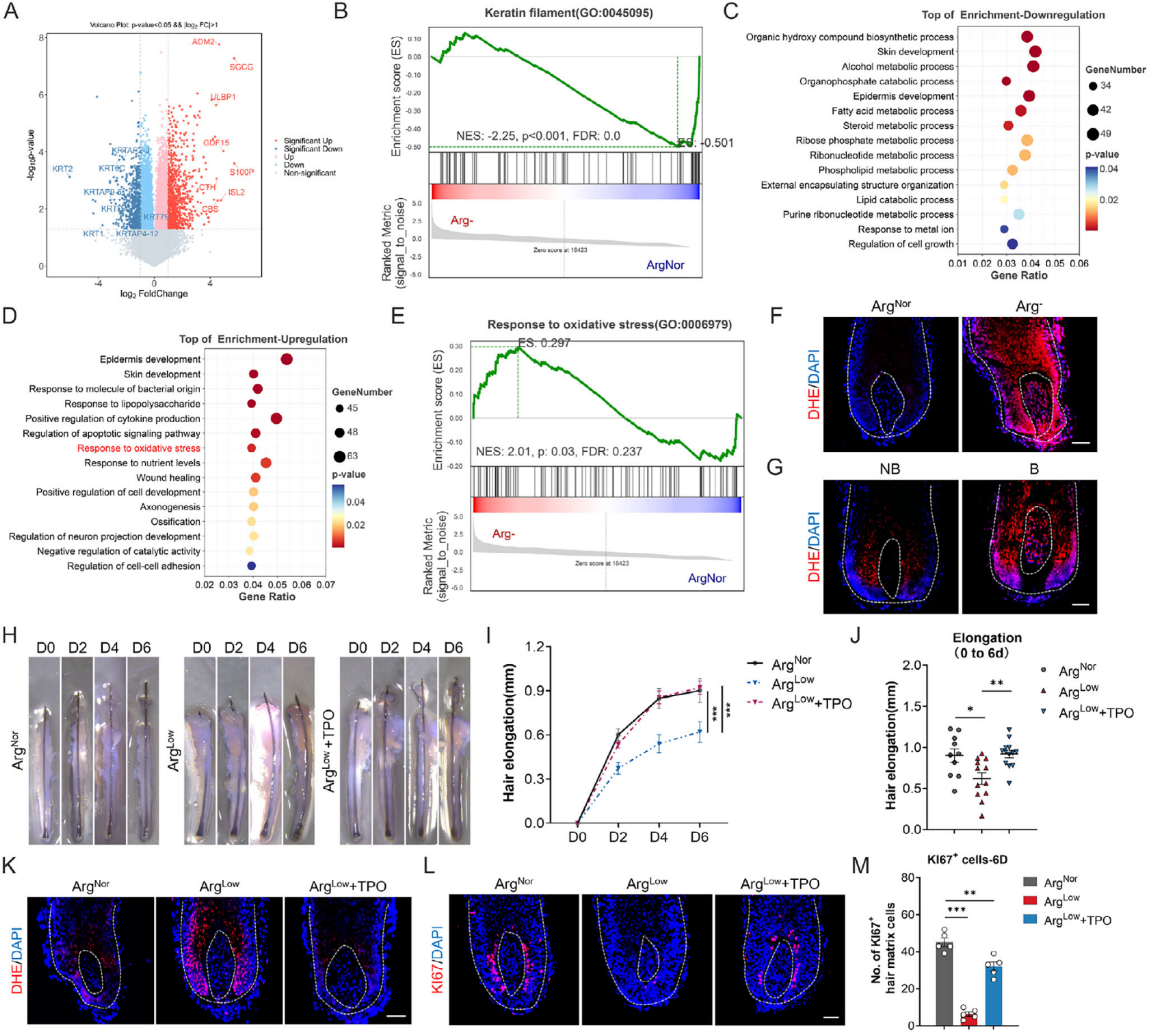
The accumulation of ROS in arginine deficiency inhibits hair growth. A) Volcano plot depicting differentially expressed genes between the control group (Arg^Nor^) and the arginine‐deficient group (Arg^−^). B) Gene Set Enrichment Analysis (GSEA) of the keratin filament pathway in arginine‐deficient hair bulbs versus control hair bulbs. GO pathway enrichment analysis showing the C) top‐scoring downregulated and D) upregulated signaling pathways in the Arg^−^ group compared to the Arg^Nor^ group. E) GSEA of the oxidative stress pathway in arginine‐deficient hair bulbs compared with control hair bulbs. F) ROS content in HFs cultured in normal medium and arginine‐deficient medium, detected using the fluorescent probe DHE. G) ROS content in HFs from non‐balding (NB) and balding (B) scalps of AGA patients, detected by DHE. H) Representative images showing hair shaft elongation in human HFs ex vivo cultured under condition of Arg^Nor^, Arg^Low^, or Arg^Low^ supplemented with 1 mm TPO. I,J) Quantification of hair shaft elongation (*n* = 12/12/13 HFs). K) ROS content in HFs (cultured as indicated) on day 2, detected by DHE. L) KI67 immunostaining of HF sections from the indicated groups. M) Quantification of the percentage of KI67^+^ matrix cells (*n* = 5 HFs per group). White curves outline representative hair bulb and DP contours. Data are presented as means ± SEM. Statistical significance was determined by J,M) one‐way ANOVA with Tukey's post hoc test and I) two‐way ANOVA with Dunnett's post hoc test. *P < 0.05, **P < 0.01, ***P < 0.001. Scale bar: 50 µm. TPO, tempol (ROS scavenger).

To elucidate arginine's regulatory mechanisms in HFs, we performed RNA‐seq on hair bulb specimens subjected to arginine deprivation. Comparative analysis revealed divergent transcriptional profiles in arginine‐deprived versus control hair bulbs (Figure S4A,B, Supporting Information). Crucially, we observed profound downregulation of multiple keratins (KRTs) and keratin‐associated proteins (KRTAPs)—critical structural determinants of hair shaft integrity. This molecular impairment was substantiated by gene set enrichment analysis (GSEA), which confirmed significant suppression of keratin filament pathway activity (**Figure** **4**A,B). Collectively, these data provide a molecular basis for the morphological aberrations in arginine‐deficient human HFs, demonstrating that arginine scarcity compromises hair keratinization.

To dissect signaling pathways mediating hair growth arrest in arginine‐deficient HFs, Gene Ontology (GO) analysis of transcriptomic profiles (Figure 4C,D) revealed marked activation of oxidative stress response pathways (Figure 4D). Consilience between GO and GSEA analyses confirmed this redox dysregulation (Figure 4D,E), while dihydroethidium (DHE) staining empirically validated pathological ROS accumulation in matrix keratinocytes—phenocopying balding HF pathology (Figure 4F,G). Complementary GSEA further demonstrated concerted enrichment of endoplasmic reticulum (ER) stress and mitochondrial dysfunction pathways (Figure S4C–E, Supporting Information), pointing to potential bioenergetic basis for ROS accrual. Direct assessment of mitochondrial integrity via JC‐1 staining established that arginine deficiency impairs mitochondrial membrane potential (Figure S4F, Supporting Information), thereby mechanistically connecting arginine depletion to mitochondrial failure and subsequent pathological ROS generation.

Building on the well‐documented regulatory function of ROS in proliferation and apoptosis, we interrogated its causal role in arginine deficiency‐induced pathology. Pharmacological ROS neutralization using the radical scavenger Tempol (TPO) rescued matrix keratinocyte proliferation and restored hair growth in arginine‐deprived conditions in cultured HF organ, with TPO's ROS clearance efficacy validated by DHE quantification (Figure 4H–M). Complementary in vitro experiments confirmed that ROS scavenging reverses arginine deficiency‐mediated cell proliferation suppression (Figure S4G–I, Supporting Information). These orthogonal functional validations establish pathological ROS accumulation as the principal mechanism executing arginine deficiency‐induced hair follicle dysfunction.

### 2.5 Arginine Deficiency Attenuates HF Growth by ROS‐Mediated mTOR Signal Suppression

**Figure 5:**
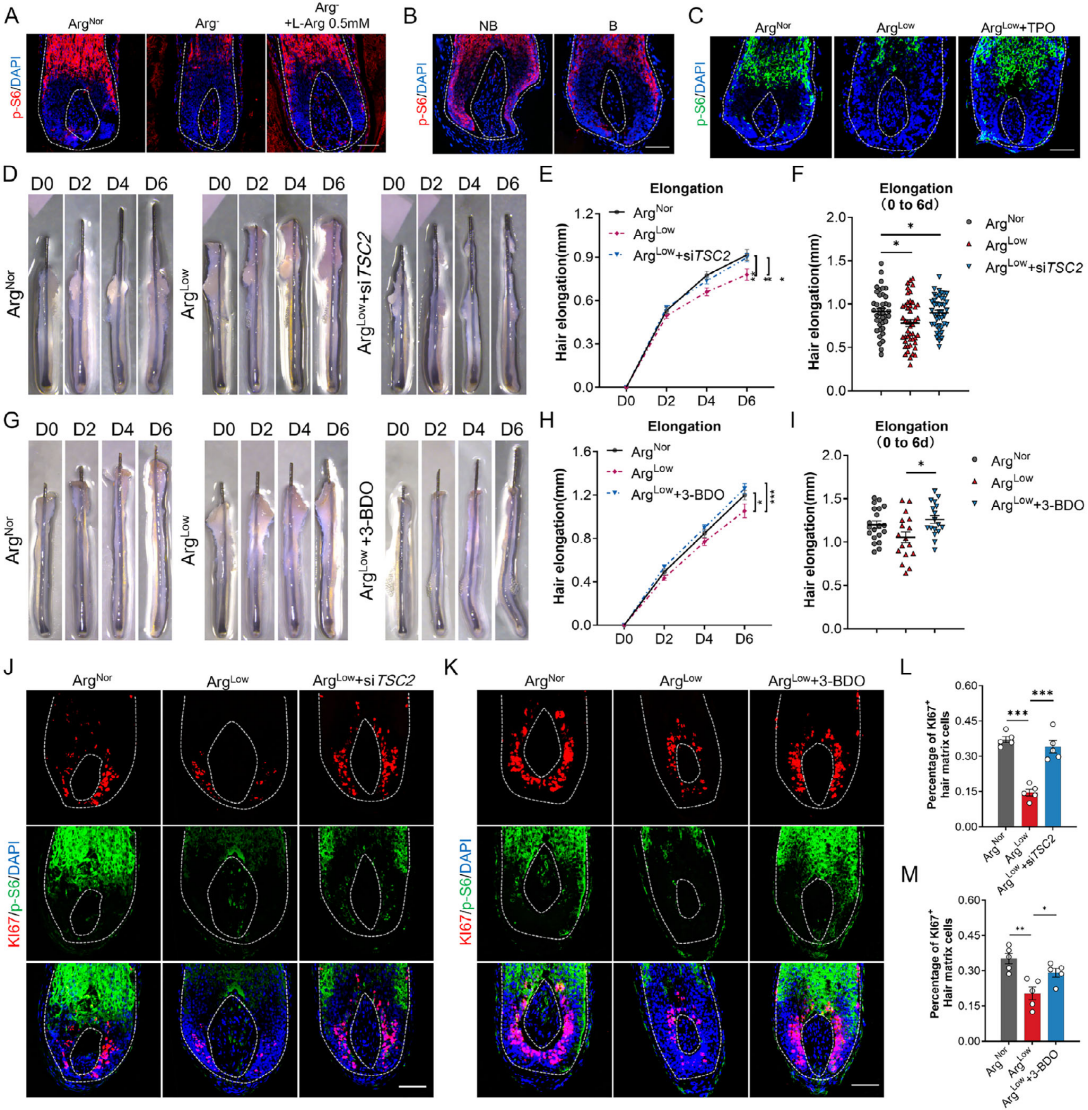
Arginine deficiency inhibits hair growth via ROS‐mediated mTOR signaling suppression. A) Phospho‐S6 (p‐S6) immunostaining in HFs cultured under normal arginine (Arg^Nor^), arginine‐deficient (Arg^−^), or Arg^−^ supplemented with 0.5 mm L‐arginine conditions. B) p‐S6 immunostaining in NB versus B scalp HFs from AGA patients. C) p‐S6 immunostaining in HFs under Arg^Nor^, Arg^Low^, orArg^Low^+TPO conditions. D) Time‐course images of HFs under arginine deprivation (to 0.05 mm) with/without siTSC2. E,F) Quantification of hair shaft elongation (*n* = 40/47/44 HFs from 3 donors). G) Representative images of HFs treated with or without arginine deprivation (to 0.05 mm) and 3‐BDO (5 µm) supplementation on days 0, 2, 4, and 6. H,I) Quantification of hair shaft elongation (*n* = 20/17/17 HFs from 3 donors). J,K) Co‐immunostaining of p‐S6 and KI67 on HF sections with indicated treatments. L,M) Quantification of the percentage of KI67^+^ matrix cells (*n* = 5 HFs per group). White curves outline representative hair bulb and DP contours (A, B, C, J, K). Data are expressed as mean ± SEM. Statistical significance was assessed by one‐way ANOVA with Tukey's post hoc test (F, I, L, M) and two‐way ANOVA with post hoc Tukey's multiple comparisons test (E, H). *p < 0.05, **p < 0.01, ***p < 0.001. Scale bar: 50 µm.

To delineate the molecular pathway linking arginine deficiency‐induced ROS accumulation to hair follicle (HF) growth suppression, we interrogated mTOR signaling—a conserved nutrient‐sensing hub engaging in bidirectional redox crosstalk.^[^ ^45^, ^46^, ^47^, ^48^, ^49^ ^]^ Assessment of ORS cells models under arginine deprivation demonstrated dose‐dependent mTOR pathway inhibition via Western blot analysis (Figure S5A, Supporting Information). Consistently, HFs cultured under arginine‐free conditions manifested mTOR suppression in hair bulb, similar to balding HFs of AGA patients (**Figure** **5**A,B). Crucially, ROS clearance using TPO reversed mTOR signaling inhibition induced by arginine deficiency (Figure 5C and Figure S5B, Supporting Information), establishing ROS as the primary mediator of mTOR dysregulation during arginine deficiency.

To further confirm the function of mTOR signaling, we administered HFs with rapamycin (RAPA), a well‐established inhibitor of mTOR, and observed its effect on hair growth. Functional validation revealed that RAPA treatment recapitulated arginine deprivation phenotypes by suppressing HF growth and matrix cell proliferation (Figure S5C–H, Supporting Information). To clarify whether the effects of arginine on hair growth are mediated through mTOR activation, we used siRNA to knock down TSC2, an endogenous inhibitor of mTORC1, to activate the mTOR pathway. TSC2 knockdown, confirmed by immunofluorescence (Figure S5I, Supporting Information), rescued arginine deficiency‐induced growth arrest while restoring mTOR signaling in cultured human HF organ, with pharmacological mTOR activation using 3‐BDO producing analogous rescue effects (Figure 5D–M).

Collectively, these findings mechanistically anchor arginine deficiency as a pathogenic driver of AGA progression through ROS‐mediated suppression of mTOR signaling—the core axis executing HF growth inhibition.

### 2.6 Arginine Exerts Beneficial Effects on Hair Growth in AGA models

**Figure 6:**
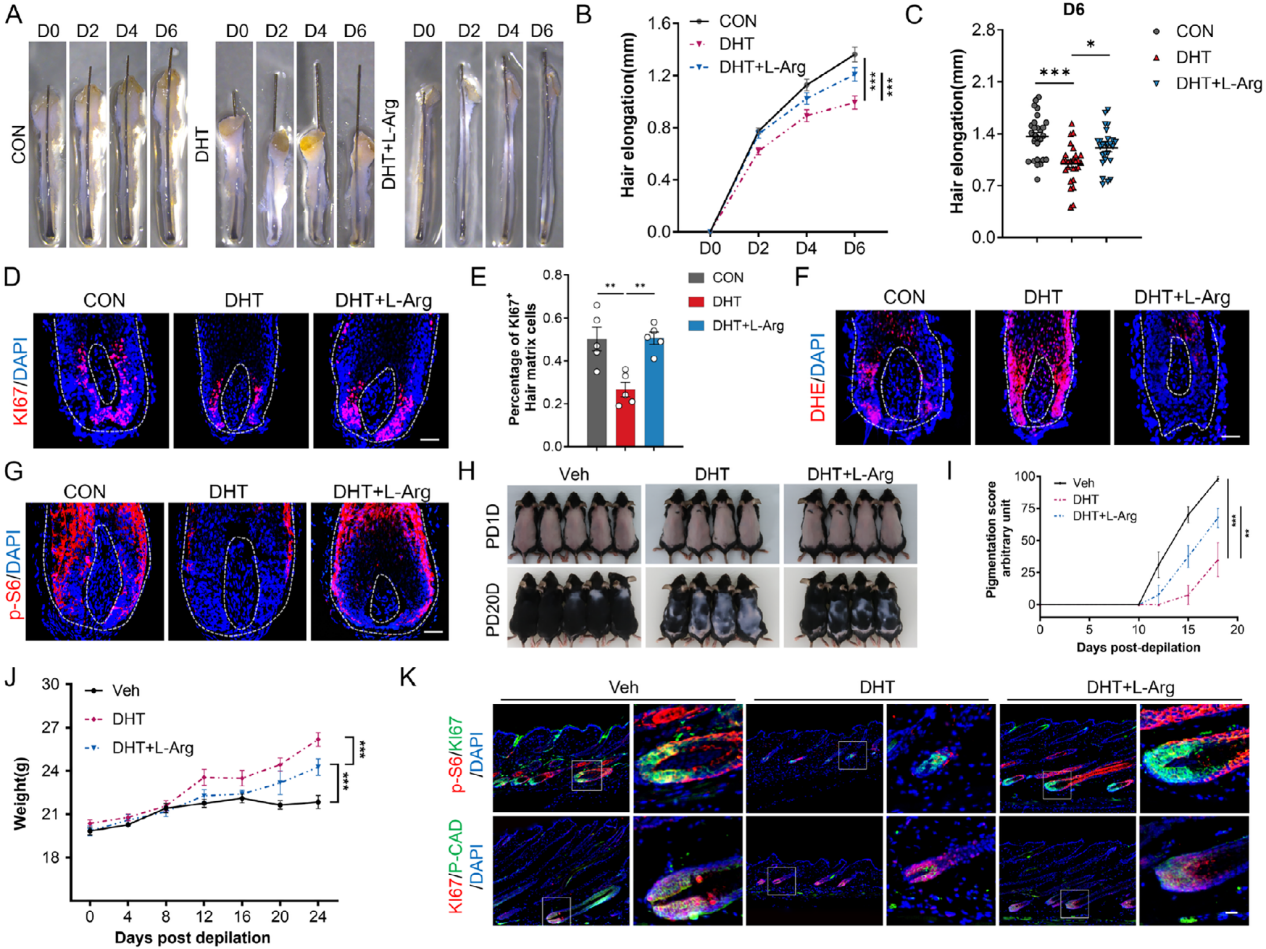
Arginine restores hair growth in DHT‐induced AGA‐like mouse model and human HFs. A) Representative time‐course images of human HFs treated with DHT (10 µM) or DHT + L‐arginine (1 mm) at days 0, 2, 4, and 6. B,C) Hair shaft elongation under control (untreated), DHT (10 µm), and DHT + L‐arginine (1 mm) treatments (*n* = 13/14/16 HFs respectively). D) KI67 immunostaining in HF sections across treatment groups. E) Quantification of the percentage of KI67^+^ matrix cells (*n* = 5 HFs per group). F) ROS content detected by the fluorescent probe DHE in HFs. G) Immunostaining of p‐S6 in HF sections. H) Hair coat regeneration in mice: Control, DHT‐induced (subcutaneous), and DHT + L‐arginine (drinking water) groups. Images captured at post‐depilation day 1 (PD1D) and day 20 (PD20D) (*n* = 5/4/4 mice). I) Hair coat recovery score following depilation (see Experimental Section). J) Body weight trajectories during treatment. K) Co‐immunostaining of p‐S6/KI67 (top) and KI67/PCAD (bottom) in mouse skin sections. The white dashed square indicates the magnified areas (K). The white dashed elliptical line delineates HF boundaries (D,F,G). Data are presented as means ± SEM. Statistical significance was determined by one‐way ANOVA with Tukey's test (C,E) and two‐way ANOVA with Dunnett's test (B,I,J). *P < 0.05, **p < 0.01, ***p < 0.001. Scale bar: 50 µm.

Although arginine's therapeutic potential in androgenetic alopecia (AGA) is recognized, its mechanistic basis remains undefined. To address this gap, we utilized dihydrotestosterone (DHT)—a core etiological factor in AGA—to establish human and murine AGA models. Both direct arginine supplementation and genetic inhibition of arginine catabolism (via ARG2 knockdown) rescued DHT‐induced hair growth suppression and matrix cell proliferation in human HFs (**Figure** **6**A–E, Figure S6A—F, Supporting Information). Consistent with AGA patient phenotypes, DHT elevated ROS while suppressing mTOR signaling in HF cells—perturbations reversed by arginine repletion (Figure 6F,G). Furthermore, oral arginine administration in DHT‐induced AGA mice yielded partial improvement in hair growth, matrix proliferation, and mTOR signaling activation (Figure 6H–K). Interestingly, while DHT injection caused an increase in body weight, oral arginine consumption mitigated this effect to some extent (Figure 6J).

**Figure 7:**
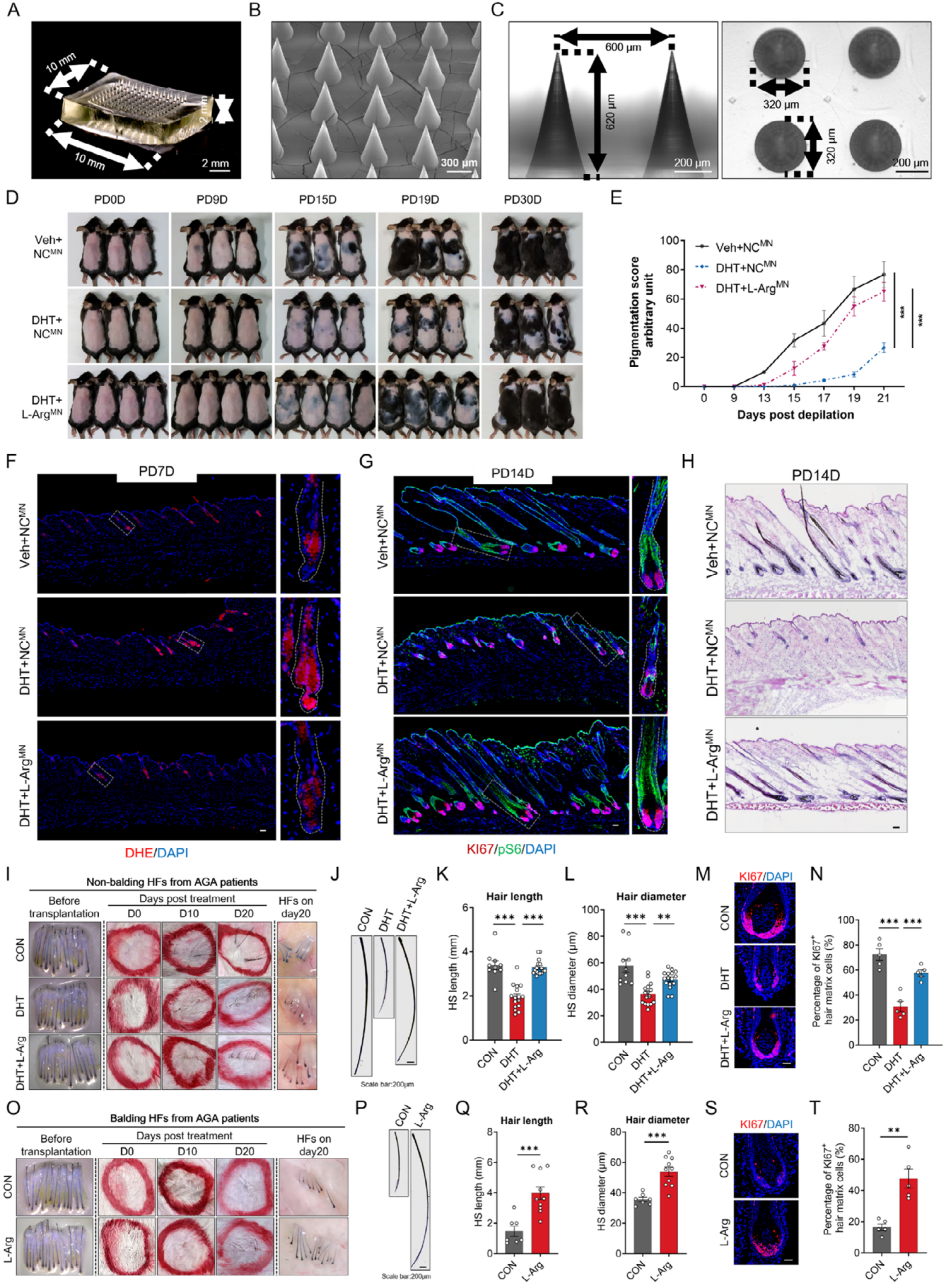
Local arginine delivery via microneedle ameliorates AGA pathology in mouse and humanized models. A) Macroscopic view of microneedle patch. Scale bar: 2 mm. B) Scanning electron microscope (SEM) images of the microneedle patch. Scale bars: 300 µm. C) Cross‐sectional (vertical) and surface (horizontal) views of microneedle patch. Scale bar: 200 µm. D) Hair coat regeneration time‐course in DHT‐induced AGA‐like mice treated with L‐arginine‐loaded microneedles (Arg^MN^). Images at post‐depilation days 0, 9, 15, 19, and 30. E Hair coat recovery scores following depilation (*n* = 10 mice per group). F) ROS content detected by DHE in dorsal skin sections. G) Co‐immunostaining of KI67 (red) and pS6 (green) in skin sections. H) H&E staining of dorsal skin at post‐depilation day 14. I–N) Non‐balding (NB) HFs from AGA patients were transplanted to SCID mice and treated with DHT alone or DHT+Arg^MN^ (DHT+L‐Arg) on day 30 post transplantation. I) Representative images of HFs before transplantation, hair growth at days 0/10/20 and HFs at day 20 post treatment. J) Representative images of hair shaft at day 20 post indicated treatment. Quantification of K) hair shaft length and L) diameter at day 20. M) KI67 staining of hair bulb at day 20. N) Quantification of KI67^+^ matrix cells percentage. O–T) Balding HFs from AGA patients were transplanted to SCID mice and treated with Arg^MN^ (L‐Arg) on day 30 post transplantation. O) Representative images of HFs before transplantation, hair growth at days 0/10/20 and HFs at day 20 post treatment. P) Representative images of hair shaft at day 20 post indicated treatment. Quantification of Q) hair shaft length and R) diameter at day 20. S) KI67 staining of hair bulb at day 20. T) Quantification of KI67^+^ matrix cells percentage. F,G) The white dashed square indicates the magnified areas and the white dashed elliptical line delineates HF boundaries. Data presented as means ± SEM. K,L,N,Q,R,T) Statistical significance was determined by one‐way ANOVA with Tukey's post hoc test two‐way ANOVA with Dunnett's post hoc test (E). Scale bar: 50 µm (unless otherwise specified).

To overcome bioavailability limitations of systemic delivery, we engineered dissolvable microneedles for targeted intradermal arginine release (**Figure** **7**A–C and Figure S7A, Supporting Information). These devices demonstrated high payload efficiency (87% w/w), effective stratum corneum penetration (axial force ≥ 0.2 N per needle), and sustained 36 h release kinetics (Figure S7B—D, Supporting Information). Upon inserting the microneedles into the mouse skin and maintaining them for 30 min, the microneedles dissolve under the influence of dermal interstitial fluid, resulting in the release of the encapsulated arginine around the dermal HFs. Methylene blue administration confirmed the microneedles’ delivery efficiency (Figure S7E, Supporting Information).

Microneedle‐mediated arginine delivery reversed DHT‐depleted dermal arginine pools (Figure S7F,G, Supporting Information), normalizing ROS accumulation and reactivating mTOR signaling to drive significant enhanced telogen‐anagen transition and hair regrowth (Figure 7D–H and Figure S7H, Supporting Information). Although monotherapy with arginine microneedles showed modest efficacy relative to minoxidil, the only FDA‐approved topical agent for AGA, combinatorial treatment demonstrated synergistic superiority over either agent alone (Figure S7I, Supporting Information). Crucially, in humanized AGA models generated by grafting AGA patient‐derived non‐balding HFs (with post‐engraftment DHT treatment) or balding HFs onto SCID mice, arginine‐loaded microneedles reversed pathognomonic hair shaft miniaturization and restored matrix proliferation (Figure 7I–T). Collectively, these results validate local arginine replenishment as a mechanistically grounded therapy for AGA.

## 3 Discussion

**Figure 8:**
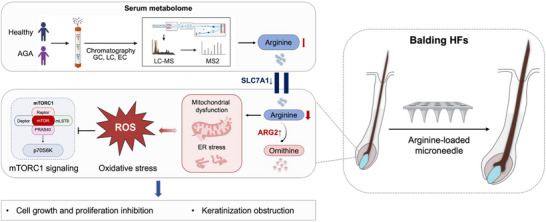
Proposed pathogenic mechanism linking impaired arginine metabolism to androgenetic alopecia (AGA) progression. Serum metabolome analysis identifies decreased circulating arginine in AGA patients. Within balding HFs, dysregulated transporters (SLC7A family) and upregulated catabolic enzyme ARG2 deplete local arginine reservoirs. This localized arginine deficiency impairs HF growth through promoting ROS accumulation and suppressing mTOR signaling, establishing it as a key driver of progression of AGA. Significantly, microneedle‐mediated local arginine supplementation reverses both hair growth inhibition and miniaturization, demonstrating therapeutic efficacy against AGA progression.

The pathogenesis of AGA represents an area of intensive research focus in recent years. Through integrative metabolomic profiling, transcriptomic analysis, and functional validation studies, our work reveals a previously unrecognized metabolic lesion in AGA: compartmentalized arginine deficiency stemming from dysregulated transport and catabolism within distinct HF microdomains. This deficiency emerges as a crucial driver of HF dysfunction via ROS/mTOR signal axis in balding HFs of AGA (**Figure** **8**).

Metabolic reprogramming constitutes a hallmark adaptation across diverse pathologies including cancer, cardiovascular disease, and diabetes.^[^ ^50^, ^51^, ^52^, ^53^, ^54^, ^55^, ^56^ ^]^ Prior comparative analyses of miniaturized versus term hairs in occipital/vertex regions similarly observed reduced amino acid/nutrient levels, aligning with our central finding of arginine deficiency in AGA‐affected follicles. However, the modest sample sizes and lack of comprehensive metabolomic profiling between healthy and AGA cohorts leave the reported metabolic alterations insufficiently substantiated.^[^ ^57^ ^]^ In the present study, we have unveiled the serum metabolic atlas of AGA patients and point to a critical metabolic imbalance, with significantly reduced arginine levels in balding HFs. We report for the first time a dysfunction in the transport and utilization of arginine in the context of AGA. Arginine can be either synthesized de novo or obtained from peripheral environment. We found that the key enzyme for synthesis ASS1 is predominantly expressed in rapidly proliferating matrix cells which is essential for HF growth. On the other hand, SLC7A1, the primary arginine transporter, is mainly expressed in HFSCs and ORS cells, which are at a lower differentiation stage. These observations suggest that different cellular population in the HFs may rely on distinct sources of arginine to support their function during the hair cycle. In contrast, ARG2, the enzyme responsible for the catabolism of arginine, is widely expressed across various HF cell populations, indicating its broad regulatory role in arginine metabolism. Actually, the upregulation of ARG2 exist in both HFSCs, ORS cells, matrix cells of balding HFs, indicating a common arginine insufficiency state in those HF cells.

In addition to arginine, our findings revealed a significant upregulation of various amino acids in the serum of AGA patients, including glutamic acid, proline, and several branched‐chain amino acids (BCAAs) such as leucine and valine. In mice, hair follicle stem cells (HFSCs) are metabolically flexible to achieve the transition to ORS progenitor cells which requires the activation of OXPHOS and flux of glutamine into TCA cycle.^[^ ^58^ ^]^ Additionally, previous research has demonstrated the ability of HFs in female pattern hair loss (FPHL) to uptake glutamic acid.^[^ ^57^ ^]^ Therefore, this warrants further in‐depth investigation in future studies to explore whether there is a disruption in the utilization of glutamine by HFs in AGA patients, which potentially leading to impaired differentiation of HFSCs. Elevated levels of BCAAs are often observed in individuals with obesity, diabetes, and metabolic syndrome, may via interfering with insulin signaling pathways.^[^ ^27^, ^59^, ^60^ ^]^ The elevation of BCAAs in AGA patients may participate in the incidence of such metabolic comorbidities, and diet restriction of such BCAAs may exert benefit for the treatment AGA.

Fundamental requirement of heightened arginine demands within actively proliferating and differentiating cellular compartments are well‐documented in prior oncology and stem cell studies.^[^ ^36^, ^58^, ^61^, ^62^, ^63^ ^]^ Our data demonstrate that within the HF niche, arginine bioavailability serve as the primary regulator governing matrix cell capacity during active HF regeneration. The premature termination of anagen, leading to hair miniaturization in balding scalps, may be partially explained by the obstruction of matrix proliferation and keratinization caused by arginine deficiency in HFs. The hair loss and CD34^+^ HFSCs reduction occurred in mice fed with Arg‐insufficient diet. Arginine has been shown to play a significant role in modulating tight junction protein expression and enhancing gap junction integrity, particularly in the context of stress and inflammation.^[^ ^64^, ^65^ ^]^ Hence, the abnormal hair shedding induced hair loss may rely on the impaired gap junctions in mice deprived of arginine. As for the reduction of CD34^+^ cells, we prefer to attributing it to the alterations of HFSC related genes expression in HFSCs as similar number of cells observed in bulge region. This change may be reversible considering the arginine function on gene expression via modulating epigenetic modification.^[^ ^66^, ^67^, ^68^ ^]^

Elevated reactive oxygen species (ROS) generated by diverse physiological stressors—including intermittent fasting, excessive polystyrene microplastic exposure, and aging—impair hair regeneration capacity.^[^ ^69^, ^70^ ^]^ While arginine was reported to regulate ROS levels through modulating nitric oxide (NO) synthesis,^[^ ^71^, ^72^ ^]^ our findings demonstrate that arginine deficiency triggers endoplasmic reticulum (ER) stress and mitochondrial dysfunction in HF cells. The ER necessitates sufficient amino acid availability for proper protein folding, while arginine scarcity compromises the synthesis of arginine‐rich proteins, thereby activating the unfolded protein response (UPR) and subsequently exacerbating ROS generation.^[^ ^73^, ^74^ ^]^ Furthermore, arginine‐modulated mitochondrial oxidative phosphorylation sustains cell proliferation during HF anagen phase,^[^ ^74^ ^]^ conversely, arginine deprivation disrupts electron transport chain flux, provoking pathological ROS accumulation. Critically, ER stress, mitochondrial dysfunction and ROS overproduction could establish a self‐amplifying vicious cycle.

mTOR integrates signals from growth factors, nutrients, and energy status to control cellular processes.^[^ ^75^ ^]^ In the presence of ROS, mTOR activity is often dysregulated, leading to impaired protein synthesis and cellular growth.^[^ ^76^, ^77^ ^]^ In addition, while streptococcal models demonstrate concomitant ROS accumulation and mTOR suppression during arginine depletion,^[^ ^78^ ^]^ direct causal validation remained absent. Our discovery that arginine deficiency inhibits the proliferation of hair matrix and, consequently, hair growth by ROS‐mediated mTOR signaling inhibition bears resemblance to observations made in cancer cells.^[^ ^79^ ^]^ Although the promotion effect of the mTOR signaling pathway on the activation of HFSCs in mice has received significant attention,^[^ ^80^ ^]^ its role in human HFs remains poorly understood. Our study revealed that the mTOR signaling pathway is primarily activated in hair bulb cells in human HFs. Of note, this activation is inhibited in balding HFs of patients with AGA and by arginine deprivation, which could lead to compromised hair growth and involved in the development of AGA.

In addition, coordinated suppression of various metabolic pathways occurs on the HFs with arginine‐depravation. Cellular metabolism provides essential energy resources, while proliferative processes require substantial bioenergetic support.^[^ ^81^ ^]^ The broad inhibition of metabolic pathways following arginine deficiency may cause bioenergetic insufficiency, ultimately constraining proliferative capacity.

Can arginine's role in promoting hair growth be exploited for AGA therapy? It has been found that the combination of inositol‐stabilized arginine silicate complex and biomagnesium can promote the regeneration of mouse nails and hair, but in this study, the use of arginine alone did not promote hair regeneration.^[^ ^82^ ^]^ In our preclinical humanized AGA model, precise localized delivery of arginine via microneedles showed a great therapeutic effect on reversing the AGA‐like hair miniaturization. The combination of Arg‐MN and minoxidil could be a powerful tool in AGA management without the side effects associated with systemic treatments.

While this study establishes compelling evidence for arginine metabolism's central role in AGA pathogenesis, several knowledge gaps persist. Our ex vivo follicle culture model successfully recapitulates short‐term physiological growth, yet its inability to maintain long‐term viability and incorporate critical niche components (e.g., immune cell, fibroblast and so on) limits investigation of microenvironmental regulation. Subsequent studies employing conditional knockout murine models will be essential to delineate the mechanistic contributions of arginine metabolism to AGA pathogenesis. Additionally, clinical trials are needed to assess the long‐term efficacy and safety of local arginine supplementation as a treatment for AGA. Nonetheless, our findings open up new possibilities for the treatment of AGA and suggest that metabolic reprogramming could be a promising strategy for managing hair loss disorders more generally.

## 4 Experimental Section

### Patient Information and Sample Collection

The participants in this study were female patients aged 18–55, diagnosed with androgenetic alopecia (AGA) by two dermatologists at the Department of Dermatology, Xiangya Hospital, from November 2019 to January 2021. The study protocol was approved by the Ethics Committee of Xiangya Hospital, Central South University. Exclusion criteria for AGA patients were as follows: 1) Not first diagnosed at Xiangya Hospital; 2) Presence of other non‐scarring alopecias such as telogen effluvium or alopecia areata; 3) Long‐term use of AGA treatments (e.g., oral antiandrogens or topical minoxidil) or other medications (e.g., corticosteroids, antihypertensive drugs, hypoglycemic agents); 4) Refusal to provide peripheral blood samples. A healthy control (HC) group, matched for age, gender, health status, and BMI, was also included. Exclusion criteria for the HC group included: 1) Long‐term use of glucocorticoids, antihypertensive drugs, or hypoglycemic agents; 2) Presence of other systemic diseases; 3) Refusal to provide peripheral blood samples.

### Human Hair Follicle Organ Culture

Hair follicle organ cultures were established using follicles micro‐dissected from AGA patients during hair transplantation surgery, with prior informed consent. HFs exhibiting intact dermal papillae and continuous dermal sheaths were isolated under stereomicroscopic guidance and cultured in 24‐well plates containing William's E‐based complete medium (supplemented with 10 mg mL^−1^ insulin, 2 mm L‐glutamine, 10 ng mL^−1^ hydrocortisone, and 100 U mL^−1^ penicillin‐streptomycin) maintained at 37 °C with 5% CO_2_ for 6–8 d. HF growth parameters were monitored by capturing images at consistent 48‐hour intervals using Leica microscopy, with subsequent quantification of elongation and diameter performed via ImageJ software.

Arginine‐deficient condition (Arg^−^) was established by replacing the basal medium with SILAC formulation (Thermo Scientific), which lacks L‐arginine, L‐leucine, and L‐lysine, followed by supplementation with 105 mg mL^−1^ L‐leucine and 146 mg mL^−1^ L‐lysine hydrochloride. The Arg^Low^ condition was generated through sub‐supplementation of the Arg^−^ medium with 0.05 mm L‐arginine.

### Animal Treatment

Female C57BL/6 mice (7 weeks old) were housed under specific‐pathogen‐free conditions (Slack Company, China) and subjected to experimental regimens following anagen induction via depilatory cream‐mediated telogen arrest. Dietary arginine manipulation utilized isocaloric diets containing normal (6.3 g per kg; Arg^Nor^) or reduced arginine (3 g per kg; Arg^Low^; Jiangsu Xietong Pharmaceutical), while systemic arginine supplementation involved ad libitum access to 7 mg mL^−1^ L‐arginine‐supplemented water. For transdermal delivery, blank (NC^MN^) or L‐arginine‐loaded microneedle patches (Arg^MN^) were applied dorsally for 60 s with subsequent 30 min dissolution. AGA mouse model was achieved through daily subcutaneous dihydrotestosterone (DHT) injections (100 µL, 0.625 mg mL^−1^; Sigma) during full hair cycles. Hair regrowth was monitored every 48 hours via quantitative assessment of skin repigmentation and hair density parameters. All protocols complied with the National Research Council's Guide and were approved by the Animal Care and Use Committee of Xiangya Hospital (IRB 201611610).

### Preparation of PVP‐Microneedles (PVP‐MNs)

PVP‐microneedle (PVP‐MN) fabrication employed polydimethylsiloxane (PDMS) molds containing 10 × 10 pyramidal cavities (base diameter: 320 µm; depth: 650 µm; pitch: 600 µm), consistent with transdermal delivery device specifications. Microneedles were produced via two‐step centrifugation casting: 50% (w/v) polyvinylpyrrolidone (PVP) solutions—either arginine‐saturated (L‐Arg^MN^) or unmodified (NC^MN^)—were introduced into molds and centrifuged (5000 rpm, 10 min) to ensure cavity infiltration, followed by blade‐mediated excess solution clearance. Following initial drying (35 °C, 6 h), a 100% (w/v) PVP solution was layered as backing material and centrifuged (5,000 rpm, 10 min) to evacuate entrapped air, with final solidification achieved through extended drying (35 °C, 12 h). Upon dermal application, rapid interstitial fluid exchange facilitated >90% microneedle dissolution within 30 minutes

### Human HF Xenograft Model

The human HF xenograft model was established following protocols^[^ ^83^ ^]^ with minor modifications. Anagen‐stage HFs from occipital (non‐balding) and vertex (balding) scalp regions of AGA patients were implanted into dorsal incisions of SCID mice (*n* = 10–20 HFs per mice) under isoflurane anesthesia. Commencing on post‐grafting day 30, cohorts received: L‐arginine‐loaded microneedles (L‐Arg), dihydrotestosterone (0.625 mg mL^−1^ DHT), or DHT + L‐Arg. Host sacrifice occurred at post‐grafting day 50. Follicle morphology and hair shaft dimensions (length/diameter) were quantified via photomicroscopy and ImageJ analysis

### Statistical Analysis

All data were expressed as mean±SEM and were analyzed by one‐way ANOVA with Tukey's post hoc test or two‐way ANOVA with Dunnett's post hoc test when more than two groups were compared or Student's t‐test when only two groups were compared. Graphpad Prism 8, R (v4.1.3) and Excel (Microsoft) were used to assess statistical significance. Statistical significance was set at a *P* < 0.05. *, ** and *** indicate *p* < 0.05, *p* < 0.01 and *p* < 0.001, respectively.

### Ethics Statement

The study protocol involving human subjects was approved by the Ethics Committee of Xiangya Hospital, Central South University (Approval No. 202203076), and conducted in accordance with the Declaration of Helsinki. Written informed consent was obtained from all participants. Animal experiments were approved by the same ethics committee (Approval No. 2022020197) and performed following institutional guidelines for laboratory animal welfare.

## Conflict of Interest

The authors declare no conflict of interest.

## Author Contributions

S.D. and G.L. contributed equally to this work. J.L., G.L., Z.D., and Y.T. designed and conceived the study. Y.T., G.L., and L.Y. performed data analyses. S.D., Y.C., and G.L. performed most experiments. Y.Z., F.L., B.W., Z.Z., and W.S. contributed to sample collection. Z.D. and M.C. help to generate sequencing libraries. H.X. and Z.W. provide critical discussion and suggestion. G.L., Y.C., Y.T., and J.L. prepared the manuscript with input from coauthors.

## Supporting information



Supporting Information

Supporting Data

## Data Availability

All data needed to assess the conclusions in the present study are provided in the manuscript and/or the Supporting Information. The RNA‐seq dataset of hair bulb with deprivation are available from the genome sequence archive under accession number HRA011571 (http://bigd.big.ac.cn/gsa‐human/). Any other data supporting the findings of this study are available from the corresponding author upon reasonable request.
