# Supplementary material for: Arginine Metabolic Disruption Impairs Hair Regeneration via ROS‐Mediated Inactivation of mTOR Signaling in Androgenetic Alopecia
Source: Adv Sci (Weinh). 2025 Aug 7;12(40):e04579. doi: 10.1002/advs.202504579 (PMC12561341; doi:10.1002/advs.202504579)
Supplement: Supplementary file 1 — Supporting Information [file ADVS-12-e04579-s001.pdf]

Supplementary materials

Supplementary methods

Targeted metabolomics sequencing and analysis

Peripheral blood (5 mL) was collected from the median cubital vein of each subject on the morning of the second day, following an overnight fast of approximately 12-16 hours since their last meal. The serum was separated and analyzed using the Q300 Kit (Metabo-Profile, China) as previously described[1]. Metabolites in the serum of AGA patients and healthy controls (HC) were quantitatively analyzed using an ultra-performance liquid chromatography coupled with tandem mass spectrometry (UPLC-MS/MS) system (ACQUITY UPLC-Xevo TQ-S, Waters Corp., Milford, MA, USA). To ensure experiment stability and sample source reliability, all tested samples were pooled to create quality control (QC) samples. One QC sample was analyzed for every 10 samples during UPLC-MS/MS analysis. MassLynx software (v4.1, Waters, Milford, MA, USA) was used to process the raw data, and IMAP (v1.0, Metabo-Profile, China) platform was used for statistical analysis, including peak integration, calibration, and quantification of each metabolite.

Assessment of Arginine Loading Efficiency in Microneedles

The UV-Vis absorption spectra of arginine aqueous solutions with varying concentrations were recorded, and a standard curve of absorbance was established at 220 nm. To determine arginine loading efficiency, 15 microneedle arrays were immersed in 1.5 mL distilled water for 10 min to ensure complete dissolution of arginine from the needle tips. During this dissolution process, polyvinylpyrrolidone K30 (PVP K30) polymer was retained using a dialysis membrane with a molecular weight cutoff of 1000 Da. The released arginine concentration in the external solution (15 mL) was periodically quantified according to the predetermined standard curve. Ultimately, the loading efficiency (%) was determined using the following equation: Loading Efficiency = (Total Released Arginine / Initial Loaded Arginine) × 100%

Mechanical Characterization of Microneedles

Previous biomechanical studies established that a minimum insertion force of 0.1 N per needle was required for microneedles to penetrate the human stratum corneum[2]. To evaluate the mechanical characterization of the fabricated microneedles, force-displacement profiles were acquired using a dynamic mechanical analyzer (DMA850, TA Instruments, USA). Quantitative analysis revealed the axial force at each needle tip (total fracture force divided by 100 needle tips) could exceed 0.2N. This measured value surpasses the established skin penetration threshold.

Immunofluorescence (IF) and TUNEL staining

Mouse skin tissue samples were harvested from mid-dorsal areas and fixed, paraffin-embedded, and cut into 5μm sections. The sections were stained with hematoxylin and

eosin (H&E) and evaluated using light microscopy (OLYMPUS, Japan). Human hair follicles were embedded in O.C.T. (Tissue Tek), and cut into 6 μm sections. For immunofluorescence, paraffin sections were deparaffinized and rehydrated and were stained with primary antibodies after antigen retrieval. The frozen sections of hair follicles were fixed with 4 % paraformaldehyde, blocked with 5 % donkey serum at room temperature for 1h, and then incubated with primary antibodies at 4 °C overnight. Alexa Fluor 488- or 594-coupled secondary antibodies (Thermo Fisher Scientific, USA) was incubated at room temperature for 1h, and 4′,6-diamidino-2-phenylindole (DAPI) was used for nuclear staining. TUNEL staining was performed according to the instruction of TUNEL assay kit (Roche, Switzerland). Photographs were captured by fluorescence microscope. Antibodys and dilution ratio are listed in TableS1.

Mouse Skin Organoid Culture

The mouse skin organoid model was established as previously described[3]. Neonatal mice, aged within 24 hours post-birth, were selected and euthanized via cervical dislocation. The back skin was carefully excised and digested with 0.1% trypsin at 4°C overnight. On the following day, the epidermis and dermis were separated and cut into small pieces. The dermal tissue was then digested with collagenase at 37°C for 30 minutes. The resulting epidermal and dermal tissue suspensions were filtered separately through a 70 μm cell strainer and centrifuged at 1,100 rpm for 15 minutes at room temperature to isolate the epidermal and dermal cell fractions. The isolated cells from both epidermis and dermis were combined and seeded into a 12-well plate containing a transwell chamber. The medium was refreshed daily, and organoid formation was monitored for 1-2 days. The abundance data for all metabolites are compiled in the spreadsheet titled " All_Metabolites" which has been uploaded as supporting data.

Arginine Detection

Arginine concentrations in serum, skin, and hair follicles were quantified using an Arginine Assay Kit (Solarbio, China), following the manufacturer's protocol. Briefly, samples were processed according to the kit instructions, and the absorbance was measured at the specified wavelength to determine the arginine concentration.

RNA extraction, real-time PCR (qPCR)

RNA was extracted from cells and human hair follicles using TRIzol Reagent (Thermo Fisher Scientific, USA) and was reverse-transcribed to cDNA by PrimeScript™ RT reagent Kit with gDNA Eraser (Takara, China). qPCR was performed on an Applied Biosystems 7500 machine (Life Technologies) with the program setted up according to the instruction of ChamQ Universal SYBR qPCR Master Mix (Vazyme, China). The relative gene expression was measured by delta-delta CT relative to GAPDH, and the fold change was normalized to the control group. The primer sequences used in this study: h*TSC2*-F: GGCAAGAGAGTAGAGAGGGACG; h*TSC2*-R: AAGAAGGG-

GGAATGGTAGAGC. h*GAPDH*-F: TGTTGCCATCAATGACCCCTT; h*GAPDH*-R: CTCCACGACGTACTCAGCG.

Immunoblotting

Cells were lysed in RIPA buffer (Thermo Fisher Scientific, USA) containing protease inhibitors (Thermo Fisher Scientific, USA) after washed with cold PBS. The proteins were quantified via bicinchoninic acid assay (Thermo Fisher Scientific, USA) and separated on SDS-PAGE and transferred to a PVDF membrane. The membrane was blocked with 5% nonfat milk for 1h at room temperature and incubated with primary antibodies overnight at 4°C. The secondary antibodies HRP-conjugated Goat anti-Mouse IgG (Santa Cruz Biotechnology, USA) and HRP-conjugated Goat anti-Rabbit IgG secondary antibody (Santa Cruz Biotechnology, USA) were incubated for 1h at room temperature. The immunoreactive bands were visualized by the HRP substrate (Sigma, USA) on ChemiDoc XRS+ system (Bio-Rad). Data were analyzed by GE Healthcare (now Cytiva) ImageQuant LAS 4000 Mini and images have been cropped for presentation. The sourced data of immunoblotting results was presented in FigS8. The primary antibodies used in this study were listed at TableS1.

RNA interference

Small interferring RNA (siRNA) were purchased from GenePharma (China). The sequences for siRNA: si*TSC2*: CAATGAGTCACAGTCCTTTGA; si*ARG2*-1: CCTATCGAGAAGGCATGTATA; si*ARG2*-2: GTTCACCAGATGAATCAGAAA; HaCaT cells were transfected with siRNA at 0.06 nM packaged by Lipofectamine 3000(Invitrogen, Carlsbad, CA, USA) in 6-well plates (5 × 105cells/well). Hair follicles were transfected with siRNA at 0.06 nM packaged by Lipofectamine 3000(Invitrogen, USA) in 24-well plates (1 hair follicle/well).

Cell culture and treatment

ORS cells were isolated from HFs of AGA patients following previously described techniques[4]. Briefly, hair follicles were collected and digested in 0.1% dispase (Sigma, USA) at 37°C for 1 hour. The connective tissue sheath (CTS) was carefully removed under a stereomicroscope, exposing the outer root sheath (ORS). The ORS was then further dissociated into individual cells by digestion with 0.05% trypsin (Gibco, USA) at 37°C for 30 minutes. The resulting cell suspension was vortexed and passed through a 40 μm cell strainer (Falcon, USA) to remove debris. The digested cells were centrifuged (300 × g, 5 min) and resuspended in culture medium. Cells were seeded onto culture plates pre-coated with 10 μg/mL human fibronectin and type I collagen (Sigma, USA) and incubated at 37°C for 1 hour. After incubation, non-adherent cells were removed by changing the culture medium. For DHT treatment experiments, cells were cultured in keratinocyte growth factor-supplemented serum-free medium (KGF-SFM) at 37°C with 5% CO_2. For arginine-manipulation studies, ORS cells were

cultured in arginine-depleted medium (following protocols for human hair follicle culture) supplemented with specified concentrations of L-Arginine (0.05mM-1mM, as indicated in experimental figures). To analyze the proliferation ability of ORS cell via colony formation, equal numbers of ORS cells (10,000) were plated in 12-well plates. After four days of cultivation, the ORS cells were fixed and stained with crystal violet (0.5% wt/vol) and photographed by Leica optical microscope.

EdU Proliferation Assay

Cell proliferation was evaluated using the EdU Cell Proliferation Assay kit (RiboBio, China). ORS cells were cultured in medium with different arginine concentration or treated with DHT or L-arginine as indicated. After 24 hours of incubation in medium containing 10 μM EdU, cells were fixed with 4% paraformaldehyde. Subsequently, cell nuclei were stained with DAPI to facilitate visualization. EdU-positive cells, indicative of DNA synthesis, were quantified using fluorescence microscopy. The proportion of EdU-incorporating cells was calculated relative to the total cell population.

Visualization of FITC-Arginine Uptake in Human HFs

To visualize the uptake of FITC-Arginine (Ruixi Biology, China), hair follicles were isolated from both balding and non-balding scalps of patients with androgenetic alopecia (AGA). The follicles were first digested in 0.1% dispase (Sigma, USA) at 37°C for 1 hour to remove the dermal sheath. The remaining hair follicles were then cultured in 24-well plates and treated with 0.5 mM FITC-Arginine for 4 hours. After culture, the hair follicles were sectioned into frozen slices, and the uptake of FITC-Arginine was visualized under a fluorescence microscope.

Oxidative Stress Detection

ROS production was measured in skin tissues, human hair follicles (HFs), and cultured cells using fluorescent probes dihydroethidium (DHE) (Beyotime, China), following the manufacturer's protocol. Fluorescent signals were captured by fluorescence microscopy, and intensity quantification was performed to determine relative ROS levels.

Reagents

L-glutamine (Gibco, USA), penicillin-streptomycin (Gibco, USA), insulin (Sigma, USA), hydrocortisone (Selleck, China), L-arginine (Sangon Biotech, China), aspartic acid (Sigma, USA), glutamic acid (Sigma, USA), alanine (Sigma, USA), L-leucine (Sigma, USA), tempol (Selleck, China), 3-BDO (Selleck, China), rapamycin (Selleck, China), and polyvinyl pyrrolidone (PVP, Aladdin, China) were used in this study.

Table

TableS1. Antibodies used in this study

**Antibody**	**Supplier**	**Purpose**	**dilution**
AR	Cell Signaling	WB	1:1000

SLC7A1	Atlas Antibodies	WB/IF	1:1000/1:100
ASS1	Proteintech	WB/IF	1:1000/1:100
ARG1	Proteintech	WB/IF	1:1000/1:100
ARG2	Proteintech	WB/IF	1:1000/1:100
Ki67	Thermo Scientific	IF	1:500
CD34	Thermo Scientific	IF	1:200
PHH3	Abcam	IF	1:500
K14	Abcam	IF	1:10000
Vimentin	Cell Signaling	IF	1:1000
TSC2	Cell Signaling	WB/IF	1:1000/1:100
p-S6	Cell Signaling	WB/IF	1:2500/1:500
S6	Cell Signaling	WB	1:1000
Gli1	Cell Signaling	WB	1:1000
p-AKT	Cell Signaling	WB	1:1000
AKT	Cell Signaling	WB	1:1000
a-β-catenin	Cell Signaling	WB	1:2000
β-catenin	Cell Signaling	WB/IF	1:1000/1:100
GAPDH	Cell Signaling	WB	1:10000
β-Tubulin	Cell Signaling	WB	1:5000
HSP90	Cell Signaling	WB	1:5000

Supplementary figures

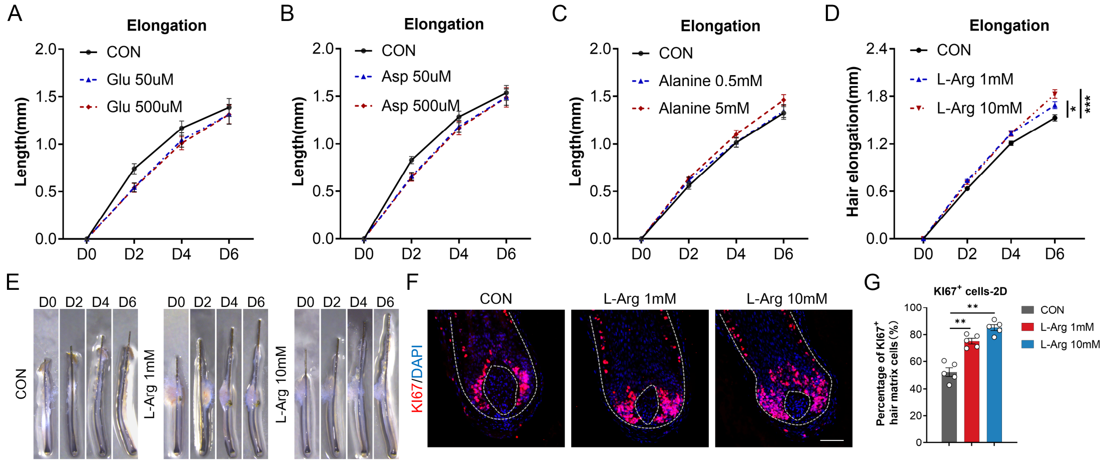

Figure S1. Supplementation with L-arginine promote HF growth (related to Figure 2).

A-D Hair elongation following treatment with alanine (n = 33/30/29 HFs), aspartic acid (n = 33/25/24 HFs, glutamic acid (n = 29/21/20 HFs) or L-arginine (n = 48/45/38 HFs). **E** Representative images of hair shaft elongation in human HFs ex vivo treated with

varying concentrations of arginine (1 mM or 10 mM). **F** Immunofluorescence staining of KI67 in HF sections. **G** Quantification of the percentage of KI67^{+} matrix cells (n = 5 HFs per group). Data are presented as means ± SEM. Statistical significance was determined by one-way ANOVA with Tukey's post hoc test (G) and two-way ANOVA with Dunnett's post hoc test (A-D). *p < 0.05, **p < 0.01, ***p < 0.001. Scale bar: 50 μm. HFs, hair follicles.

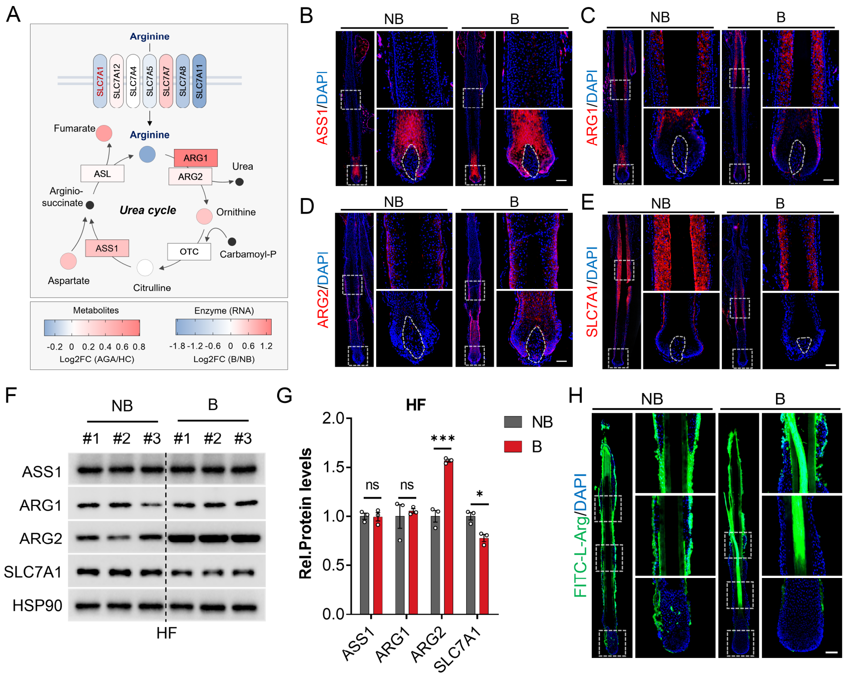

Figure S2. Arginine metabolism is aberrant in AGA patients (Related to Figure 2). **A** Differential expression of enzymes, transporters, and metabolites in the arginine metabolism pathway. Enzymes are represented by rectangles, transporters by rounded rectangles, and metabolites by circles. Red indicates upregulation, blue indicates downregulation, and black indicates metabolites not detected. **B-E** Immunofluorescence staining of ASS1 (B), ARG1 (C), ARG2 (D), and SLC7A1 (E) in anagen HFs from NB/B scalps of AGA patients. **F-G** Protein expression levels of ASS1, ARG1, ARG2, and SLC7A1 in HFs from NB/B scalp regions of AGA patients, measured by western blotting (n = 3 individuals). **H** Fluorescence microscopy images showing the uptake of FITC-labeled arginine (FITC-Arg) in HFs from NB/B scalps of AGA patients. The white dashed square indicates the magnified area and the white dashed elliptical line indicates dermal papilla (B, C, D, E, H). Data are presented as means ± SEM. Statistical significance was determined by two-tailed unpaired Student's t-test (G). *P < 0.05, **P < 0.01, ***P < 0.001. Scale bar: 50 μm. NB, non-balding; B, balding; ASS1, argininosuccinate synthetase 1; ASL, argininosuccinate lyase; ARG1,

arginase 1; ARG2, arginase 2; SLC7A1/5/8/11, solute carrier family 7 members 1/5/8/11.

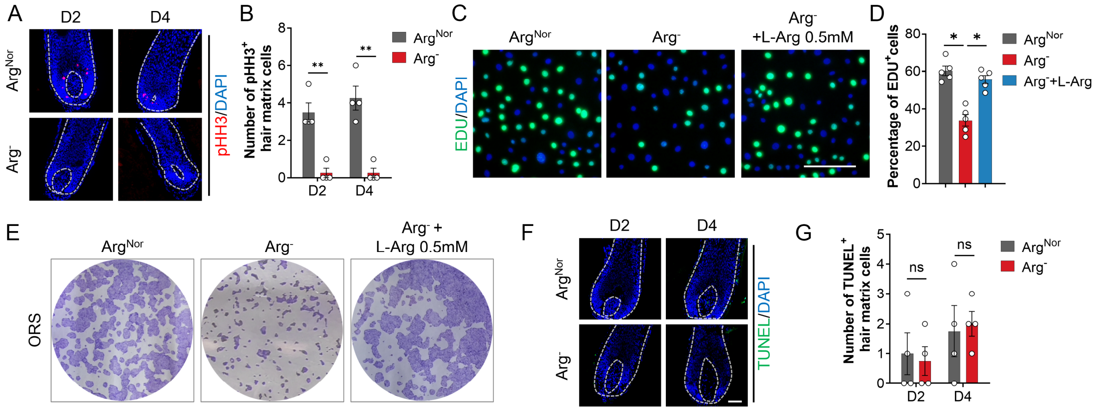

Figure S3. Arginine promotes cell proliferation of HF cells (related to Figure 2). **A** Immunostaining of phosphorylated histone H3 (pHH3) in HF sections. **B** Quantification of the number of pHH3+ matrix cells (n = 3-4 HFs per group). **C** EdU staining of outer root sheath (ORS) cells with indicated treatments for 48 hours. **D** Quantification of the percentage of EdU^{+} ORS cells (n = 5 fields per group). **E** Representative colonies formed by ORS cells cultured in Arg^{Nor}, Arg–, or Arg– + L-Arg (0.5 mM) medium for 4 days. **F** TUNEL staining of HF sections. **G** Quantification of the number of TUNEL+ matrix cells (n = 4 HFs per group). The white curve outlines the general contour of the selected hair bulb and DP (A, F). Data are expressed as mean ± SEM. Statistical significance was assessed by two-tailed unpaired Student's t-test (B, G) and one-way ANOVA with Tukey's post hoc test (D). *p < 0.05, **p < 0.01, ***p < 0.001. Scale bar :50 μm.

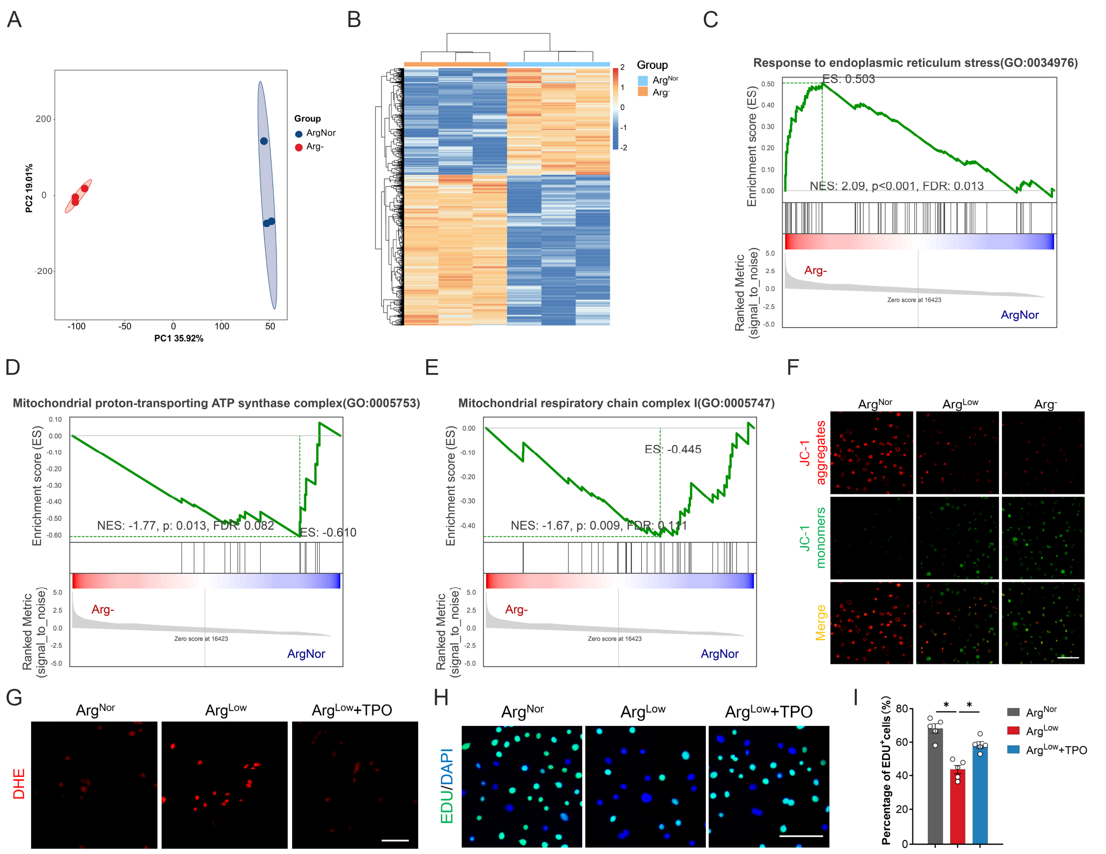

Figure S4. Transcriptome profiling identified differentially expressed genes and signal pathways in hair bulbs under arginine-deficient conditions (related to Figure 4).

A Principal component analysis (PCA) of differences between the control (Arg^{Nor}) and arginine-deficient (Arg^{-}) groups. **B** Heatmap showing differentially expressed genes between the two groups. **C-E** GSEA analysis of indicated signals in Arg^{Nor} and Arg^{-} groups. **F** JC-1 staining of ORS cells cultured in medium with different arginine concentration. **G** ROS production in ORS cells cultured in the indicated medium for 48 hours, detected by the DHE fluorescent probe. **H** EdU staining of ORS cells cultured in medium of Arg^{Nor}, Arg^{Low}, or Arg^{Low} supplemented with 1 mM tempol (TPO) for 48 hours. **I** Quantification of the percentage of EdU^{+} ORS cells (n = 5 fields per group). Data are presented as means ± SEM. Statistical significance was determined by one-way ANOVA with Tukey's post hoc test (I). *$p < 0.05$, **$p < 0.01$, ***$p < 0.001$. Scale bar: 50 μm. TPO, tempol (ROS scavenger).

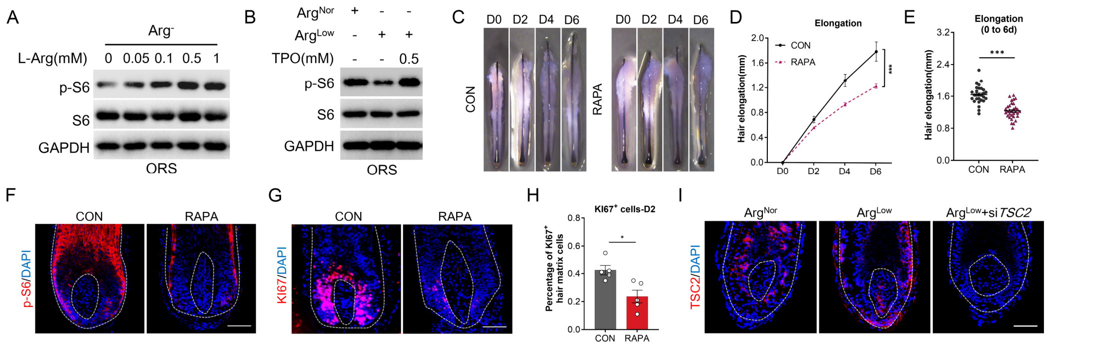

Figure S5. mTOR signaling suppression inhibits hair growth (Related to Figure 5). **A** Western blot analysis of protein levels of p-S6, S6 in ORS cells cultured with varying concentrations of arginine for 48 hours. **B** Immunostaining of p-S6 on HF sections from NB and B scalps of AGA patients. **C** Representative images of HFs treated with rapamycin (RAPA, 50 μM) on days 0, 2, 4, and 6. **D, E** Elongation of hair shafts following treatment (n = 21/25 HFs). **F, G** Immunostaining of p-S6 (F) and KI67 (G) in HF sections. **H** Quantification of $KI67^{+}$ matrix cells (n = 3-5 HFs per group). **I** Immunostaining of TSC2 in HF sections with indicated treatments. The white dashed elliptical line indicates the hair bulb and dermal papilla (F, G, I). Data are expressed as mean ± SEM. Statistical significance was assessed by two-tailed unpaired Student's t-test (E, H) and two-way ANOVA with Dunnett's post hoc test (D). *p < 0.05, **p < 0.01, ***p < 0.001. Scale bar: 50 μm

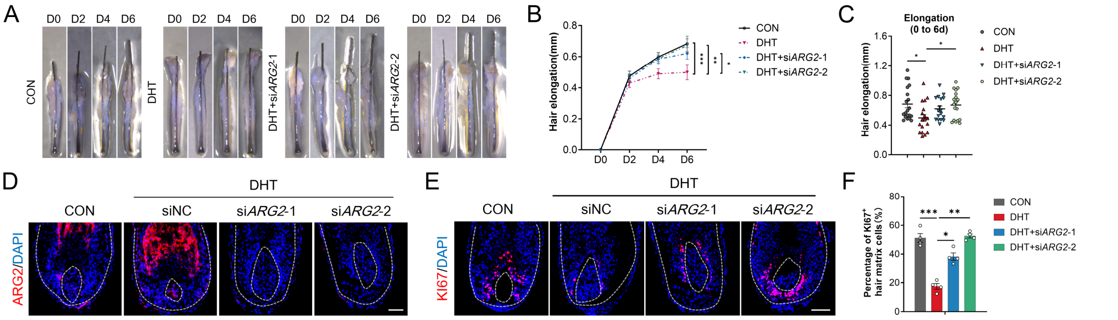

Figure S6. Knockdown of *ARG2* restored DHT-induced HF growth inhibition (Related to Figure 6).

A Representative image of HFs treated with DHT (10 μM) or DHT combined with si*ARG2* interference on days 0, 2, 4, and 6. **B, C** Elongation of the hair shaft (n = 18/17/18/18 HFs). **D, E** Immunostaining of ARG2 (D) or KI67 (E) on HF sections. **F** Quantification of the percentage of $KI67^{+}$ matrix cells (n = 4 HFs per group). Statistical significance was determined by one-way ANOVA with Tukey's post hoc test (C, F) and two-way ANOVA with Dunnett's post hoc test (B). *p < 0.05, **p < 0.01, ***p < 0.001. Scale bar: 50 μm

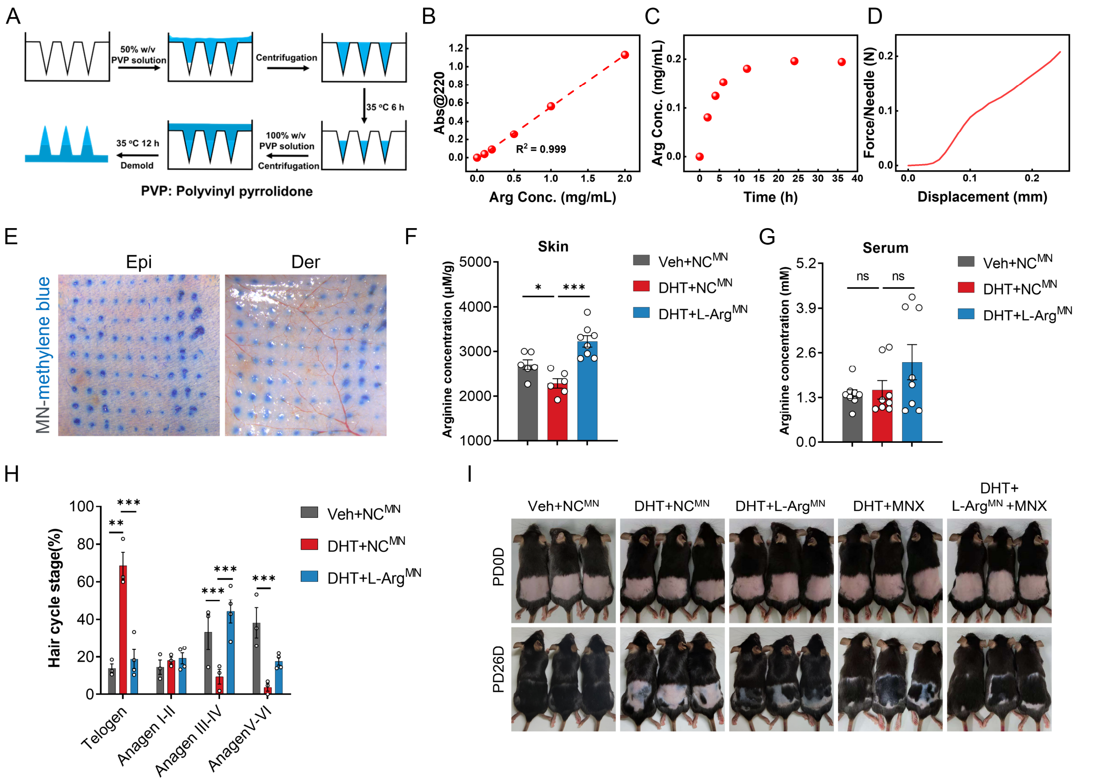

Figure S7. Characterization and functional evaluation of arginine-loaded microneedles (Arg^{MN}) (Related to Figure 7).

A Schematic fabrication of Arg^{MN}. **B** Standard curve of absorbance at 220 nm for L-arginine quantification. **C** Arginine release kinetics from arginine-loaded microneedles over time. **D** Force-displacement curve of arginine-loaded microneedles during mechanical compression. **E** Transdermal distribution of methylene blue tracer delivered by microneedles. **F,G** Skin tissue (F) and serum (G) arginine concentrations in mice treated with Arg^{MN} vs. controls (n = 6 skin samples/group; n = 8 serum samples/group). **H** Hair cycle stage quantification at postnatal day 14 (n = 3/3/4 mice). **K** Representative images of the hair coats of mice treated with Arg^{MN}/minoxidil on their dorsal skin, combined with subcutaneous DHT injection. Data are presented as means ± SEM. Statistical significance was determined by one-way ANOVA with Tukey's post hoc test (F, G) and two-way ANOVA with Dunnett's post hoc test (H). *$p < 0.05$, **$p < 0.01$, ***$p < 0.001$. Scale bar: 50 μm

Figure S2F

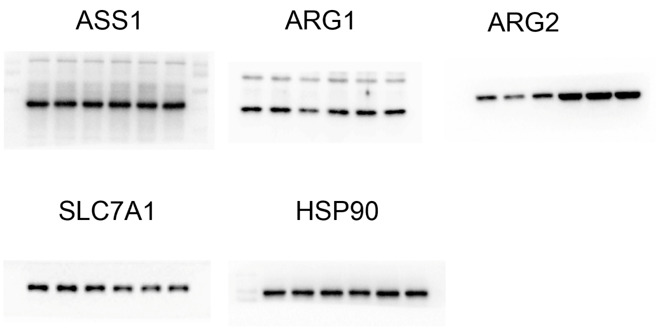

Figure S5A

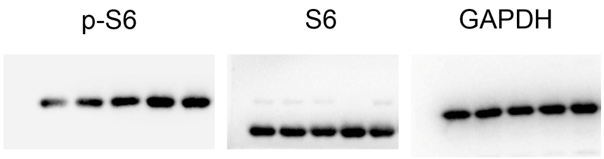

Figure S5D

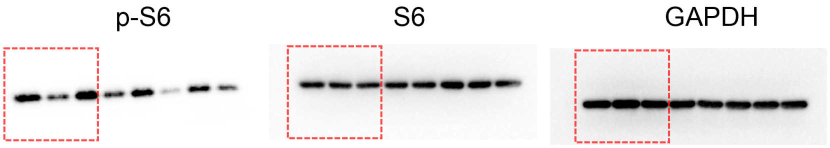

Figure S8. Sourced data of WB results in Figure S2F, Figure S5A and Figure S5D.
